# Supplementary material for: Wafer-scale manufacturing of ultra-broadband, high-power erbium-doped integrated lasers
Source: Nat Commun. 2026 Mar 10;17:3722. doi: 10.1038/s41467-026-69787-1 (PMC13102962; doi:10.1038/s41467-026-69787-1)
Supplement: Supplementary file 1 — Supplementary Information [file 41467_2026_69787_MOESM1_ESM.pdf]

Supplementary Information for: Wafer-scale manufacturing of ultra-broadband, high-power erbium-doped integrated lasers

Xinru Ji[1,2], Xuan Yang[1,2], Yang Liu[1,2], Zheru Qiu[1,2], Grigory Lihachev[1,2,4], Simone Bianconi[1,2], Jiale Sun[1,2], Andrey Voloshin[1,2], Taegon Kim[3], Joseph C. Olson[3], and Tobias J. Kippenberg[1,2†]

[1] *Institute of Physics, Swiss Federal Institute of Technology Lausanne (EPFL), CH-1015 Lausanne, Switzerland*

[2] *Institute of Electrical and Micro Engineering, Swiss Federal Institute of Technology Lausanne (EPFL), CH-1015 Lausanne, Switzerland*

[3] *Varian Semiconductor, Applied Materials, Gloucester, MA 01930, United States*

[4] *EDWATEC, CH-1015 Lausanne, Switzerland*

This Supplementary Information presents a comparative study of ion implantation in thick and thin Er-doped Si_3N_4 waveguides, the impact of annealing-etching sequence and edge-coupler design, and the modeling and characterization of broadband tunable loop mirrors and Vernier filters. It further provides a travelling-wave analysis of EDWL output power and Schawlow–Townes linewidth, and quantifies frequency-noise contributions from thermorefractive noise, pump laser RIN, heater resistivity drift, external injection, and self-reflection, including the roles of hydrogen-related absorption and Er^{3+} quenching. Finally, we assess wafer-scale uniformity, amplifier noise figure, footprint scaling strategies, lasing in high-Er-concentration devices, and refractive-index modification of Si_3N_4 by Er implantation.

Contents

Supplementary Note 1. Ion implantation parameters for Er-doped Si_3N_4 devices: thick vs. thin waveguides 3

Supplementary Note 2. Impact of annealing and etching sequence on Si_3N_4 waveguides 4

Supplementary Note 3. Broadband tunable loop mirror design and characterization 5

Supplementary Note 4. Edge coupler characterization 7

Supplementary Note 5. Vernier filter transmission analysis 7

Supplementary Note 6. Theoretical analysis of the EDWL output power and linewidth 8

Supplementary Note 7. Frequency noise transduction from thermorefractive noise in Vernier ring resonators 13

Supplementary Note 8. Frequency noise transduction from pump laser RIN 15

Supplementary Note 9. Wafer-scale uniformity of the EDWL tunability and frequency noise 16

Supplementary Note 10. EDWL frequency noise response to external injection and self-reflection 16

Supplementary Note 11. Hydrogen-related absorption and Er^{3+} quenching in Si_3N_4 PICs 19

Supplementary Note 12. Group delay of Vernier resonators 19

Supplementary Note 13. Effect of heater resistivity drift on laser frequency stability 20

Supplementary Note 14. Noise figure measurement of erbium-doped waveguide amplifiers 21

Supplementary Note 15. Footprint considerations and compact layout 21

Supplementary Note 16. Lasing in high-Er-concentration EDWLs 22

Supplementary Note 17. Refractive index modification of Si_3N_4 by Er implantation 23

Supplementary Note 1. Ion implantation parameters for Er-doped Si_3N_4 devices: thick vs. thin waveguides

Supplementary Table S1. Comparison of fabrication requirements and ion implantation parameters for thick (700 nm) and thin (200 nm) Er-doped Si_3N_4 devices

Si_3N_4 waveguide thickness (nm)	Fabrication steps for passive waveguides	Implantation machine model	Ion energy (keV)	Ion fluence (cm^{-2})	Beam current density (cm^{-2})	Implantation area (cm^2)	Net time
200	4*	VIISta HE	480	3.20×10^{15}	0.15 μA	~707 (12″ wafer)	60 min.
			270	1.50×10^{15}	0.15 μA		30 min.
			130	1.10×10^{15}	0.3 μA		10 min.
700	6**	Tandem	2000	4.50×10^{15}	13.8 – 304.5 pA	~0.72	18 hours
			1416	3.17×10^{15}	41.5 – 152.2 pA		19 hours
			955	2.34×10^{15}	96.9 – 179.9 pA		9.5 hours

* 4 steps: Deposition, patterning, etching, and annealing of the Si_3N_4 waveguides [1].
** 6 steps: Patterning, preform etching, preform reflow, deposition, planarization, and annealing of the Si_3N_4 waveguides [2].

In the development of erbium (Er) doped silicon nitride (Si_3N_4) waveguide devices, the choice of ion implantation parameters—particularly ion energy and dose—plays a critical role in determining the feasibility and scalability of the fabrication process. In our prior works [3, 4], thick silicon nitride waveguides (~700 nm) with high confinement required high-energy ion implantations up to 2 MeV to achieve optimal optical mode overlap with the erbium ions. However, such high-energy implantations are not commonly used in industrial applications due to their high cost and limited accessibility, especially in large-scale processes. Additionally, high-energy implants typically require advanced equipment setups like gas-insulated electrostatic accelerators, which further increase operational complexity. To address these challenges, there is a shift towards using thinner silicon nitride layers (~200 nm), allowing for ion implantation at significantly lower energies, around 480 keV. This reduction in ion energy aligns more closely with the implantation parameters used in current microelectronics manufacturing, where standard implanters typically operate below 600 keV for most high-dose applications [5].

Supplementary Table S1 summarizes key Er ion implantation parameters from our recent runs, including fabrication steps for passive waveguide preparation, ion energy, fluence, beam current density, and net implantation time, which generally scales with ion fluence. The 200 nm waveguide height is carefully chosen to optimize the overlap with Er ions while aligning with available implantation energies in the semiconductor industry.

A comparison of Er absorption profiles, characterized by intrinsic linewidths in Er:Si_3N_4 microring resonators, is provided in Supplementary Figure S1(a)(b). Supplementary Figure S1(a) shows the intrinsic linewidth ($\kappa_0/2\pi$) of a 2 MeV implanted resonator with a 2.1×0.7 μm^2 cross-section and 100 GHz FSR, while Supplementary Figure S1(b) depicts the intrinsic linewidth of a 480 keV implanted resonator with a 5×0.2 μm^2 cross-section and 50 GHz FSR. Implantation parameters for both resonators are detailed in Supplementary Table S1. $\kappa_0/2\pi$, measured via frequency-comb-assisted broadband laser spectroscopy, reveal characteristic Er absorption profiles in both high-energy and low-energy samples. Differences in peak absorption (maximum of $\kappa_0/2\pi$) arise from variations in Er profiles optimized for overlap factors. By scaling the Er ion implantation dose in Supplementary Figure S1(b) to match the peak absorption in Supplementary Figure S1(a), similar peak absorption can be achieved in the low-energy implanted sample. However, in thinner Si_3N_4 waveguides, closer Er-ion spacing compared to thicker waveguides increases the likelihood of ion clustering at high implantation doses. Elevated Er concentrations further induce pair-induced quenching, caused by energy transfer between closely spaced ions, which hinders full population inversion and reduces quantum efficiency [6, 7]. To mitigate these effects while maintaining high gain and efficiency, implantation parameters for 200 nm Si_3N_4 waveguides are carefully optimized.

By moving towards lower-energy implantations, we aim to achieve greater availability and economic viability, enabling wafer-scale doping of Si_3N_4 waveguides (up to 12″ wafers with the VIISta HE implanter, Supplementary Figure S1(d)), compared to the previous high-energy approach, which was typically limited to small-area doping (~8.5×8.5 mm^2 with the Tandem implanter, Supplementary Figure S1(c)). The VIISta HE implanter provides a dose uniformity within 0.5% (1σ) and an angle accuracy of $\pm0.1°$ for 8-inch wafers. The shift to thinner nitride films

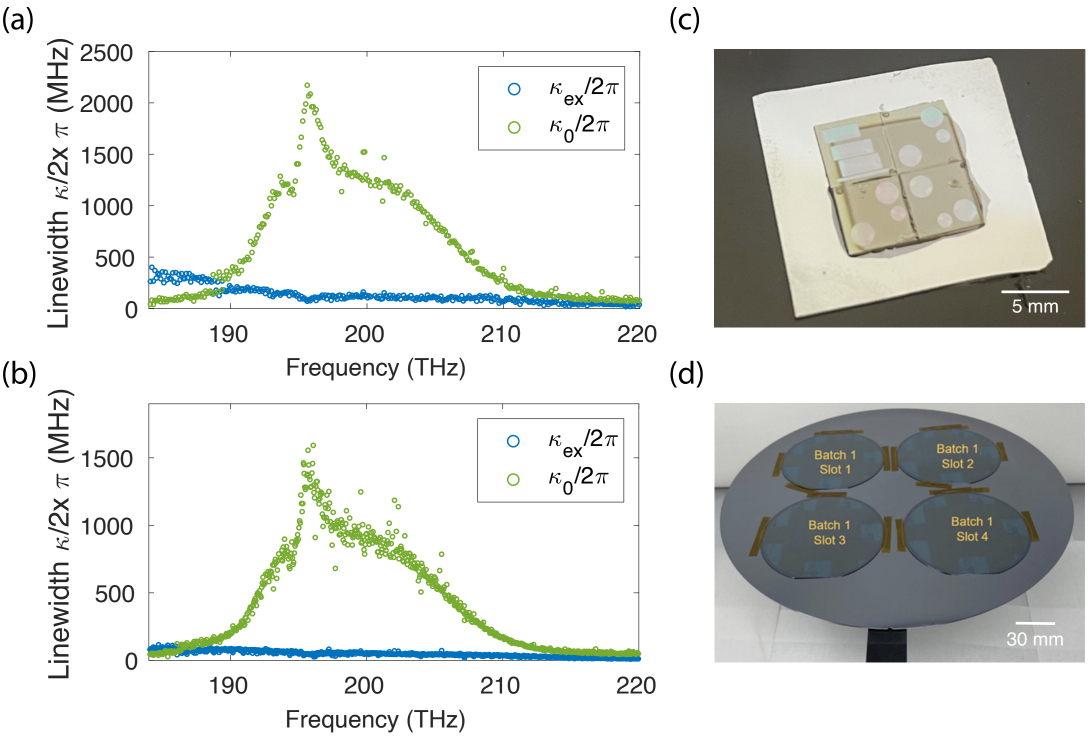

Supplementary Figure S1. **Comparison of high-energy and low-energy Er implantations** (**a**) Measured optical losses (intrinsic linewidths $\kappa_0/2\pi$) of the Er-implanted Si_3N_4 microring resonator with 2 MeV Er implantation energy. The implantation parameters are given in Supplementary Table S1. The microring resonator features a cross-section of 2.1×0.7 μm^2 and a free spectral range (FSR) of 100 GHz. The intrinsic linewidths present a characteristic Er absorption profile. (**b**) Measured optical losses (intrinsic linewidths $\kappa_0/2\pi$) of the Er-implanted Si_3N_4 microring resonator with 480 keV Er implantation energy, using the implantation parameters given in Supplementary Table S1. The Si_3N_4 waveguide has a cross-section of 5×0.2 μm^2 and an FSR of 50 GHz. (**c**) Picture of the high-energy implanted samples characterized in (**a**). The total implantation area is 8.5×8.5 mm^2. (**d**) Picture of the low-energy-implanted samples characterized in (**b**). This picture shows four 4-inch wafers mounted on a 12-inch wafer during implantation. The total implantation area is up to 707 cm^2, corresponding to a 12$''$ wafer.

and lower-energy implantations not only improves cost-efficiency but also enhances device throughput by leveraging high-current and medium-current implanters already prevalent in the semiconductor industry. Aligning with these established protocols makes the fabrication of Er-doped Si_3N_4 devices more scalable and commercially viable for practical applications.

Supplementary Note 2. Impact of annealing and etching sequence on Si_3N_4 waveguides

We investigated the impact of the etching-annealing sequence on passive Si_3N_4 waveguides.

In the fabrication process for the EDWL devices discussed in the main manuscript, 200 nm LPCVD Si_3N_4 is deposited on a silicon substrate with an 8 μm wet oxide (WOX) layer, followed by a high-temperature annealing. Depending on the conditions, high-temperature annealing generally induces film shrinkage in LPCVD Si_3N_4 films [8]. Here, we anneal the Si_3N_4 film at 1200 °C for 11 hours, necessitating an investigation into the resulting thickness reduction. We measured a 6.8 nm average thickness reduction in a 200 nm Si_3N_4 film after annealing (Supplementary Figure S2(b)). Such shrinkage can significantly affect devices that are sensitive to waveguide height variations. For instance, in waveguide Bragg gratings, a thickness variation of 7 nm can cause a central reflection frequency shift on the order of terahertz.

The intrinsic tensile stress of LPCVD Si_3N_4 films causes unpredictable bending in 5 μm $\times$ 200 nm waveguides following the standard etching-annealing process [1, 9], as illustrated in Supplementary Figure S2(c,d). This bending affects the optical mode distribution and can lead to additional scattering loss, which may impact the performance of the erbium-doped gain section in the EDWL devices. However, reversing the sequence of Si_3N_4 waveguide etching and annealing effectively mitigates this bending. Supplementary Figure S2(e) presents the cross-section of a Si_3N_4

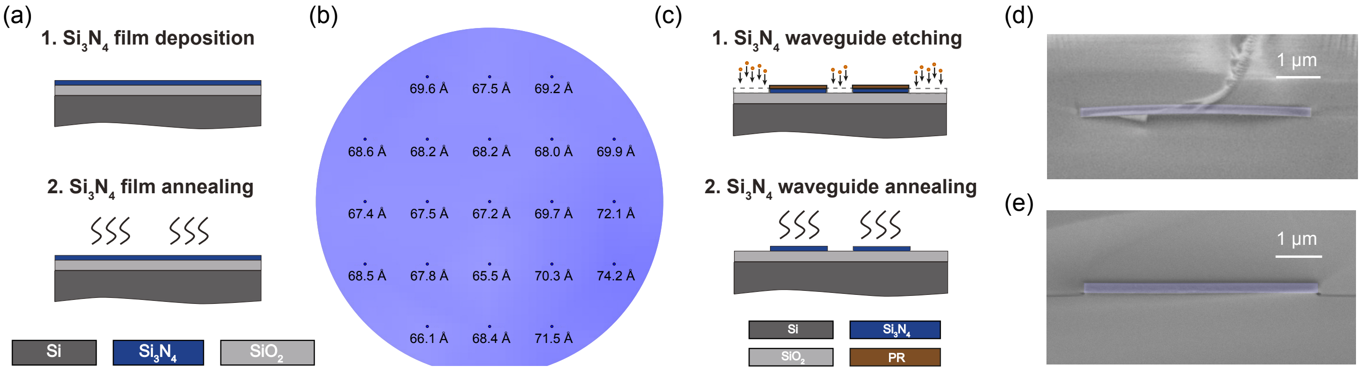

Supplementary Figure S2. **Effect of annealing-etching order on Si_3N_4 waveguide properties.** (**a**) Pre-fabrication of Si_3N_4 waveguides: Low-pressure chemical vapor deposition of Si_3N_4 thin films and film annealing at 1200 °C for 11 hours. (**b**) Shrinkage of a 200 nm Si_3N_4 film thickness after annealing. (**c**) Schematic of Si_3N_4 waveguide fabrication process, showing etching and annealing steps. (**d**) The scanning electron microscopic image of Si_3N_4 waveguide fabricated with etching-annealing sequence. (**e**) The scanning electron microscopic image of Si_3N_4 waveguide fabricated with annealing-etching sequence.

waveguide fabricated with the annealing-etching sequence, where no bending is observed, preserving a rectangular cross-sectional shape. The annealing-etching sequence also allows improved control of waveguide thickness, as it facilitates thickness measurement at the film level.

Supplementary Note 3. Broadband tunable loop mirror design and characterization

The tunable broadband loop mirror used in the EDWL in the main text, illustrated in Supplementary Figure S3, is based on a looped Mach-Zehnder Interferometer (MZI) structure. The device features two input fields, E_{i1} and E_{i2}, and two output fields, E_{o1} and E_{o2}. Directional couplers within the MZI, defined by coupling ratios k_1 and k_2, govern the power distribution between the two arms. Phase tuning is implemented using integrated metal heaters, which introduce controlled phase shifts to dynamically adjust the transmission and reflection characteristics of the loop mirror, enabling broadband tuning of the output fields E_{o1} and E_{o2}.

The mathematical relationship between E_{o1}, E_{o2} and E_{i1}, E_{i2} is written as:

$$
\begin{aligned}
\begin{bmatrix} E_{\text{o1}} \\ E_{\text{o2}} \end{bmatrix} = {} & \begin{bmatrix} \sqrt{1-k_1} & -i\sqrt{k_1} \\ -i\sqrt{k_1} & \sqrt{1-k_1} \end{bmatrix} \times \begin{bmatrix} \exp\left(-i\beta L_1 - i\Delta\varphi\right) & 0 \\ 0 & \exp\left(-i\beta L_2\right) \end{bmatrix} \\
& \times \begin{bmatrix} \sqrt{1-k_2} & -i\sqrt{k_2} \\ -i\sqrt{k_2} & \sqrt{1-k_2} \end{bmatrix} \times \begin{bmatrix} \exp(-i\beta L) & 0 \\ 0 & \exp(-i\beta L) \end{bmatrix} \\
& \times \text{ swap } \times \begin{bmatrix} \sqrt{1-k_2} & -i\sqrt{k_2} \\ -i\sqrt{k_2} & \sqrt{1-k_2} \end{bmatrix} \times \begin{bmatrix} \exp\left(-i\beta L_1 - i\Delta\varphi\right) & 0 \\ 0 & \exp\left(-i\beta L_2\right) \end{bmatrix} \\
& \times \begin{bmatrix} \sqrt{1-k_1} & -i\sqrt{k_1} \\ -i\sqrt{k_1} & \sqrt{1-k_1} \end{bmatrix} \times \begin{bmatrix} E_{\text{i1}} \\ E_{\text{i2}} \end{bmatrix}
\end{aligned} \tag{1}
$$

The operator swap interchanges the components of the vector it acts upon, such that:

$$
\text{swap} \times \begin{bmatrix} A \\ B \end{bmatrix} = \begin{bmatrix} B \\ A \end{bmatrix} \tag{2}
$$

The output fields E_{o1}, E_{o2} are computed as:

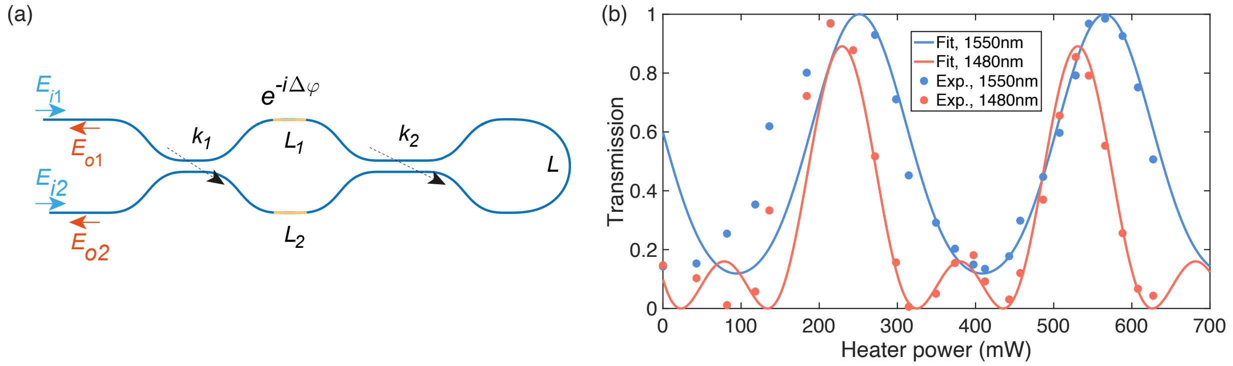

Supplementary Figure S3. **Broadband tunable loop mirror characteristics.** (**a**) Schematic of the tunable loop mirror. E_{i1} and E_{i2}: input fields; E_{o1} and E_{o2}: output fields; k_1 and k_2: power coupling ratios in the directional couplers; L_1 and L_2: lengths of the Mach-Zehnder Interferometer (MZI) arms. (**b**) Simulated and experimental dependence of transmitted output field on applied heater power in the MZI arm. Simulation parameters for 1550 nm: E_{i1}=0, E_{i2}=1, k_1 = k_2=0.09; for 1480 nm: E_{i1}=0, E_{i2}=1, k_1=0.16, k_2=0.3.

$$
\begin{aligned}
E_{o1} = &\left(-ie^{-i\Delta\varphi}\sqrt{1-k_1}\sqrt{k_2} - i\sqrt{k_1}\sqrt{1-k_2}\right)\\
&\times\left[E_0\left(-\sqrt{k_1}\sqrt{k_2} + e^{-i\Delta\varphi}\sqrt{1-k_1}\sqrt{1-k_2}\right) + E_1\left(-ie^{-i\Delta\varphi}\sqrt{k_1}\sqrt{1-k_2} - i\sqrt{1-k_1}\sqrt{k_2}\right)\right]\\
&+\left(-\sqrt{k_1}\sqrt{k_2} + e^{-i\Delta\varphi}\sqrt{1-k_1}\sqrt{1-k_2}\right)\\
&\times\left[E_0\left(-ie^{-i\Delta\varphi}\sqrt{1-k_1}\sqrt{k_2} - i\sqrt{k_1}\sqrt{1-k_2}\right) + E_1\left(\sqrt{1-k_1}\sqrt{1-k_2} - e^{-i\Delta\varphi}\sqrt{k_1}\sqrt{k_2}\right)\right]\\
E_{o2} = &\left(-ie^{-i\Delta\varphi}\sqrt{k_1}\sqrt{1-k_2} - i\sqrt{1-k_1}\sqrt{k_2}\right)\\
&\times\left[E_0\left(-ie^{-i\Delta\varphi}\sqrt{1-k_1}\sqrt{k_2} - i\sqrt{k_1}\sqrt{1-k_2}\right) + E_1\left(\sqrt{1-k_1}\sqrt{1-k_2} - e^{-i\Delta\varphi}\sqrt{k_1}\sqrt{k_2}\right)\right]\\
&+\left(\sqrt{1-k_1}\sqrt{1-k_2} - e^{-i\Delta\varphi}\sqrt{k_1}\sqrt{k_2}\right)\\
&\times\left[E_0\left(-\sqrt{k_1}\sqrt{k_2} + e^{-i\Delta\varphi}\sqrt{1-k_1}\sqrt{1-k_2}\right) + E_1\left(-ie^{-i\Delta\varphi}\sqrt{k_1}\sqrt{1-k_2} - i\sqrt{1-k_1}\sqrt{k_2}\right)\right]
\end{aligned}
\tag{3}
$$

In Supplementary Equations 1 and 3, β is the propagation constant, and $e^{-i\Delta\varphi}$ denotes the additional phase shift introduced by micro-heaters. The nonlinear phase shift over the closed loop of length L is neglected, and the MZI arm lengths L_1 and L_2 are set equal.

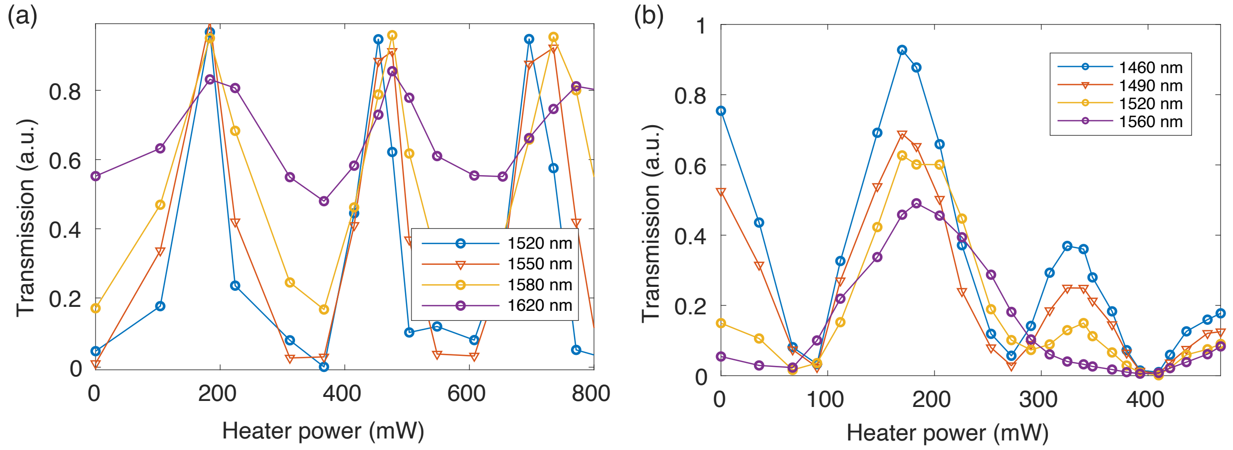

Supplementary Figure S4. **Heater power-dependent tunable loop mirror characterization.** Transmission of (**a**) the broadband reflector and (**b**) mode-selective reflector in the EDWL.

Supplementary Figure S3(b) presents experimental and simulated transmission characteristics as a function of applied heater power in a tunable loop mirror for 1480 nm and 1550 nm inputs. In these measurements, the input field is injected at one port (E_{i1}=0 and E_{i2}=1), and transmission is defined as the output at the opposite port ($|E_{o1}|^2$). For both wavelengths, transmission is tunable from 0 to 1, demonstrating the loop mirror's ability to switch between fully transmissive and fully reflective states by adjusting heater power. Deviations at lower heater powers arise from resistivity fluctuations due to heat dissipation and environmental influences.

Adjusting the power coupling ratios k_1 and k_2 in the directional couplers, along with applying appropriate heater powers (e.g., ∼600 mW as in Supplementary Figure S3(b)), enables opposite output behavior for the 1480 nm and 1550 nm inputs, effectively serving as a wavelength division multiplexer.

In the design of the broadband tunable loop mirrors for the EDWL device, two reflectors were engineered with distinct functionalities to optimize laser performance. The output reflector provides tunable broadband reflectivity for lasing wavelengths, while the other reflects C- and L-band lasing and transmits 1480 nm Er pump light. These functionalities were achieved by tailoring the coupling ratios k_1 and k_2 in the directional couplers. Both mirrors are tunable via a bias applied to the MZI arms.

Supplementary Figure S4 presents the experimental characterization of the tunable loop mirrors. By varying the heater voltage, transmission was measured and normalized to a reference waveguide, with polarization set to transverse electric (TE) mode. The results demonstrate tunable broadband reflection (Supplementary Figure S4(a)) for the EDWL output power modulation, and tunable mode-selective reflection (Supplementary Figure S4(b), heater power at 0 mW or >300 mW) for Er ion excitation and lasing mode reflection.

Supplementary Note 4. Edge coupler characterization

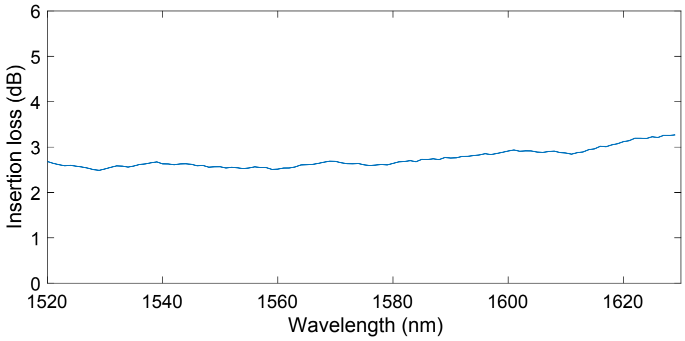

Supplementary Figure S5. **Insertion loss (fiber-to-fiber) through a reference waveguide with UHNA-7 fibers.**

Insertion loss was measured on a reference waveguide with 0.5-μm-wide, 200-nm-thick edge couplers identical to the output edge coupler of our EDWL. In Supplementary Figure S5, the measured fiber-to-fiber insertion loss at 1550 nm is 2.566 dB (1.28 dB per facet), varying between 2.48 dB and 3.26 dB over the 1520–1630 nm wavelength range. Simulations for the EDWL coupler predict ∼0.56 dB per facet at 1550 nm. The discrepancy is attributed to fabrication-induced taper width/height deviations (including tip erosion) and small alignment errors that increase overlap loss. Further reducing the UHNA-7 to SMF-28 splice loss should also narrow this gap.

Supplementary Note 5. Vernier filter transmission analysis

We present the simulation and measurement of the Vernier-filter transmission. The Vernier filter transmission, $T_\mathrm{f} = |s_\mathrm{out}|^2$, is given by (see Eq. 12, derived in Section Supplementary Note 7):

$$T_\mathrm{f} = \left| \frac{\kappa_\mathrm{ex,1} \cdot \kappa_\mathrm{ex,2}}{(i\Delta_1 + \kappa_1) \cdot (i\Delta_2 + \kappa_2)} \right|^2, \kappa_\mathrm{j} = \frac{\kappa_0}{2} + \kappa_\mathrm{ex,j} + \kappa_\mathrm{p,j} \; (j = 1, 2) \tag{4}$$

where Δ_j is the detuning of resonator j, $\kappa_\mathrm{ex,j}(\lambda)$ are the external coupling rates, $\kappa_0(\lambda)$ is the intrinsic loss rate, and $\kappa_\mathrm{p,j}$ account for parasitic coupling (higher-order and radiation modes).

In Supplementary Figure S6(a), we measure the wavelength-dependent κ_ex using a test resonator that shares the Vernier geometry but employs a single bus coupling with a 2 μm gap. Vernier resonator κ_ex values are then calibrated from the simulated κ_ex-gap relation (Lumerical FDTD).

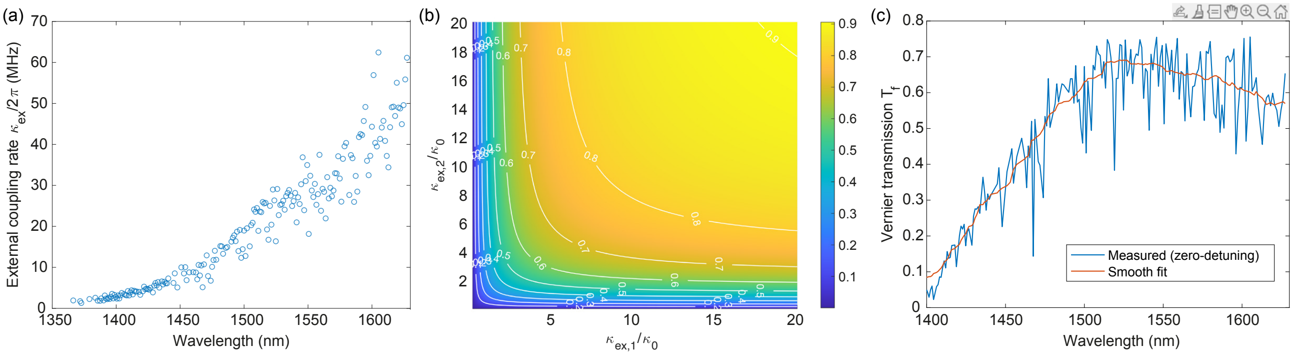

Supplementary Figure S6. **Vernier filter loss analysis.** (**a**) Measured external coupling rate $\kappa_{\mathrm{ex}}/2\pi$ versus wavelength for a test ring resonator sharing the Vernier ring geometry but using a single bus waveguide and a 2 μm gap. Vernier ring κ_{ex} values are calibrated via a κ_{ex}-gap relation simulated using Lumerical FDTD. (**b**) Simulated Vernier filter transmission as a function of $\kappa_{\mathrm{ex}}/\kappa_0$ for both resonators, evaluated at zero detuning. (**c**) Fitted transmission of Vernier filter in the EDWL.

Supplementary Figure S6(b) shows the simulated Vernier transmission versus the normalized coupling ($\kappa_{\mathrm{ex,j}}/\kappa_0$): increasing ($\kappa_{\mathrm{ex,j}}/\kappa_0$) for both resonators raises the peak filter transmission. Using the calibrated κ_{ex} and the κ_0 from Fig. 2D, Supplementary Figure S6(c) shows Vernier filter transmission at different wavelengths, yielding 0.6–0.7 in the C and L bands. Higher transmission is attainable with larger κ_{ex} (smaller gaps) or reduced κ_0.

Supplementary Note 6. Theoretical analysis of the EDWL output power and linewidth

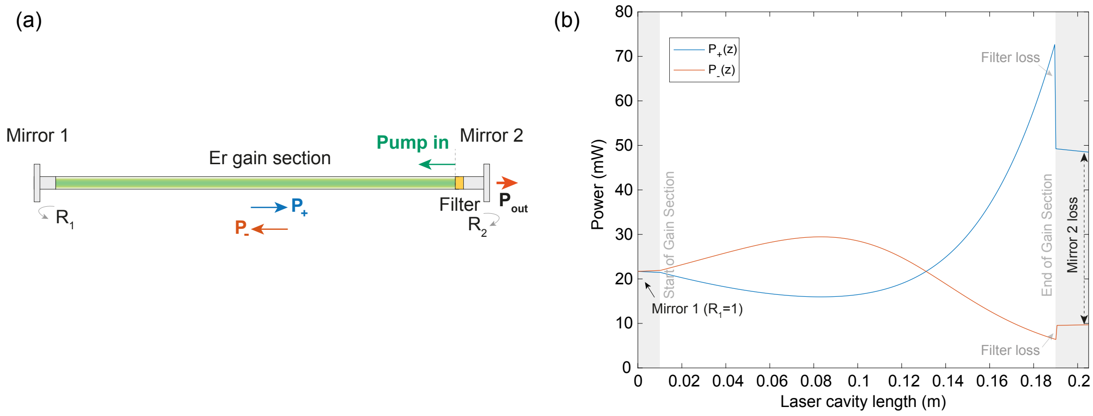

Supplementary Figure S7. **Simulation of intra-cavity power distribution in an EDWL.** (**a**) Schematic of the laser cavity, featuring two mirrors, an erbium-doped gain section, and a mode filter. Simulated output power is collected from mirror 2. The pump light is injected in the backward direction between the Er-doped gain section and the Vernier filter. (**b**) Simulated intra-cavity power distribution for forward (P_+) and backward (P_-) propagating light. Losses due to mirror transmission and filtering are highlighted.

This section investigates the theoretical performance of the EDWL by simulating the output power and fundamental linewidth using typical parameters presented in this work.

The rate equation governing the complex light field $A(t)$ within a single-mode laser cavity is expressed as:

$$\frac{\mathrm{n_g} L}{c} \cdot \frac{\partial A}{\partial t} = \left(\frac{gL}{2} - \frac{\alpha L}{2}\right) A(t) - \left[\ln\left(\frac{1}{\sqrt{R_1}}\right) + \ln\left(\frac{1}{\sqrt{R_2}}\right) + \ln\left(\frac{1}{T_{\mathrm{f}}}\right)\right] A(t) \tag{5}$$

where $\mathrm{n_g}$ is the group index, L is the cavity round-trip length (~40.14 cm), g is the gain coefficient (1/m), and α is the propagation loss (1/m). The term $\mathrm{n_g}L/c$ represents the round-trip time, measured as 3.33 ns from the longitudinal mode spacing. This exceeds the calculated value by 0.92 ns due to group delay from the Vernier filter microresonators, as discussed in the following sections.

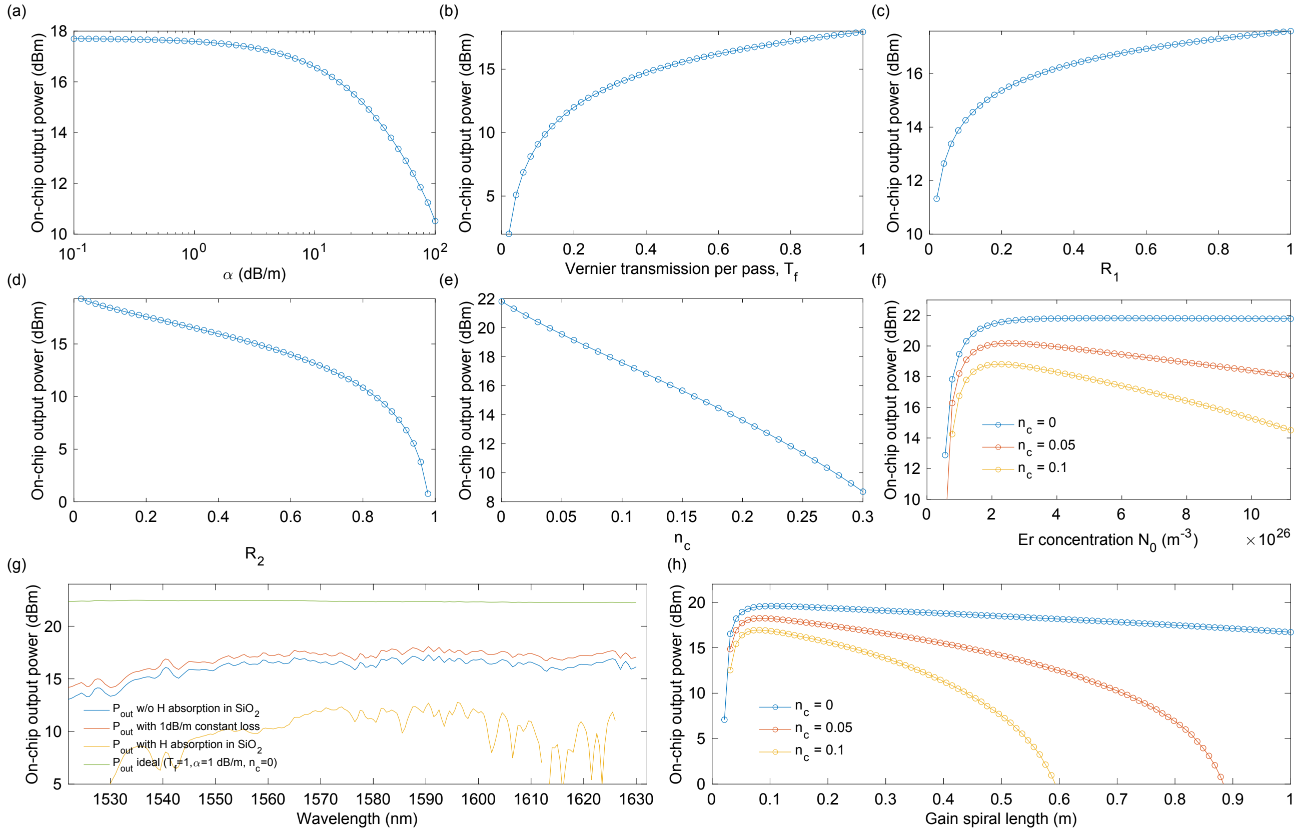

Supplementary Figure S8. **Parametric sensitivity of the output power P_{out} at 1550 nm, at a fixed on-chip pump power of 200 mW.** (**a**) Output power as a function of passive loss α (dB/m) with Vernier filter transmission T_{f}=0.9, R_1=1, R_2=0.2, non-emitting (parasitic) fraction n_{c}=0.1. The pump loss is fixed to 1 dB/m. (**b**) Output power as a function of Vernier filter transmission T_{f}, with α=1 dB/m, R_1=1, R_2=0.2, n_{c}=0.1. (**c**) Output power as a function of R_1, with α=1 dB/m, T_{f}=0.9, R_2=0.2, n_{c}=0.1. (**d**) Output power as a function of R_2, with α=1 dB/m, T_{f}=0.9, R_1=1, n_{c}=0.1. (**e**) Output power as a function of non-emitting ion fraction n_{c}, with α=1 dB/m, T_{f}=0.9, R_1=1, R_2=0.2. (**f**) Output power as a function of Er concentration N_0, with α=1 dB/m, T_{f}=0.9, R_1=1, R_2=0.2, for n_{c}=0, 0.05 and 0.1. (**a**–**f**) assume the implanted Er concentration N_0=5.6×10^{26} m^{-3}. (**g**) Simulated wavelength-dependent output power, based on measured passive loss and Vernier transmission from fabricated devices (blue trace: R_1=1, R_2=0.2, n_{c}=0.1, N_0=5.6×10^{26} m^{-3}), comparing with a constant propagation loss of 1 dB/m (red trace). This simulation also considers SiO_2 cladding with H-related absorption near 200 THz (yellow trace, with pump loss of 20 dB/m), and an ideal case (green trace, α=1 dB/m, R_1=1, R_2=0.2, T_{f}=1, n_{c}=0.1, N_0=5.6×10^{26} m^{-3}). (**h**) Output power as a function of gain spiral length, based on measured passive loss and Vernier transmission from fabricated devices, showing an optimum around 0.08–0.1 m at an Er concentration of N_0=5.6×10^{26} m^{-3}, with n_{c} varying from 0 to 0.1.

At steady state, $\mathrm{d}A/\mathrm{d}t = 0$, the round-trip field equation indicates gain saturation $g = \alpha + \frac{2}{L}\ln\frac{1}{\sqrt{R_1}\sqrt{R_2}T_{\mathrm{f}}}$: As intracavity power increases, the effective gain g decreases until it equals the total cavity loss, leading to steady-state gain clamping.

In Eq. 5, $A(t)$ denotes the circulating field amplitude averaged over one cavity round trip, providing a lumped description of the temporal field evolution. To capture spatial effects such as gain depletion and power variation along the erbium-doped waveguide, the model is extended to a distributed form, $A(z,t)$, implemented through a travelling-wave, self-consistent Er^{3+} rate equation ($^4\mathrm{I}_{13/2} \leftrightarrow {}^4\mathrm{I}_{15/2}$) solver that propagates the forward and backward pump and signal:

$$\begin{aligned}
\frac{\mathrm{d}N_2}{\mathrm{d}t} &= -\frac{N_2}{\tau} + \left(N_1\sigma_{\mathrm{s},12} - N_2\sigma_{\mathrm{s},21}\right)\phi_{\mathrm{s}} + \left(N_1\sigma_{\mathrm{p},12} - N_2\sigma_{\mathrm{p},21}\right)\phi_{\mathrm{p}} \\
\frac{\mathrm{d}N_1}{\mathrm{d}t} &= \frac{N_2}{\tau} + \left(N_2\sigma_{\mathrm{s},21} - N_1\sigma_{\mathrm{s},12}\right)\phi_{\mathrm{s}} + \left(N_2\sigma_{\mathrm{p},21} - N_1\sigma_{\mathrm{p},12}\right)\phi_{\mathrm{p}}
\end{aligned} \tag{6}$$

where $\sigma_{\mathrm{s},21}, \sigma_{\mathrm{p},21}$ and $\sigma_{\mathrm{s},12}, \sigma_{\mathrm{p},12}$ are the emission and absorption cross sections (taken from [3]), ϕ_{s}, ϕ_{p} denote the ion flux, with $\phi_{\mathrm{s}} = \frac{\mathrm{I_s}}{h\nu_{\mathrm{s}}}$ and $\phi_{\mathrm{p}} = \frac{I_{\mathrm{p}}}{h\nu_{\mathrm{p}}}$. I_{s} and I_{p} are ion-weighted intensities and are calculated as $I_{\mathrm{s}} = \Gamma_{\mathrm{s}}\frac{P_{\mathrm{s}}}{A_{\mathrm{s,eff}}}$, and $I_{\mathrm{p}} = \Gamma_{\mathrm{p}}\frac{P_{\mathrm{p}}}{A_{\mathrm{p,eff}}}$, considering that only the portion of the optical field overlapping with the doped region contributes to

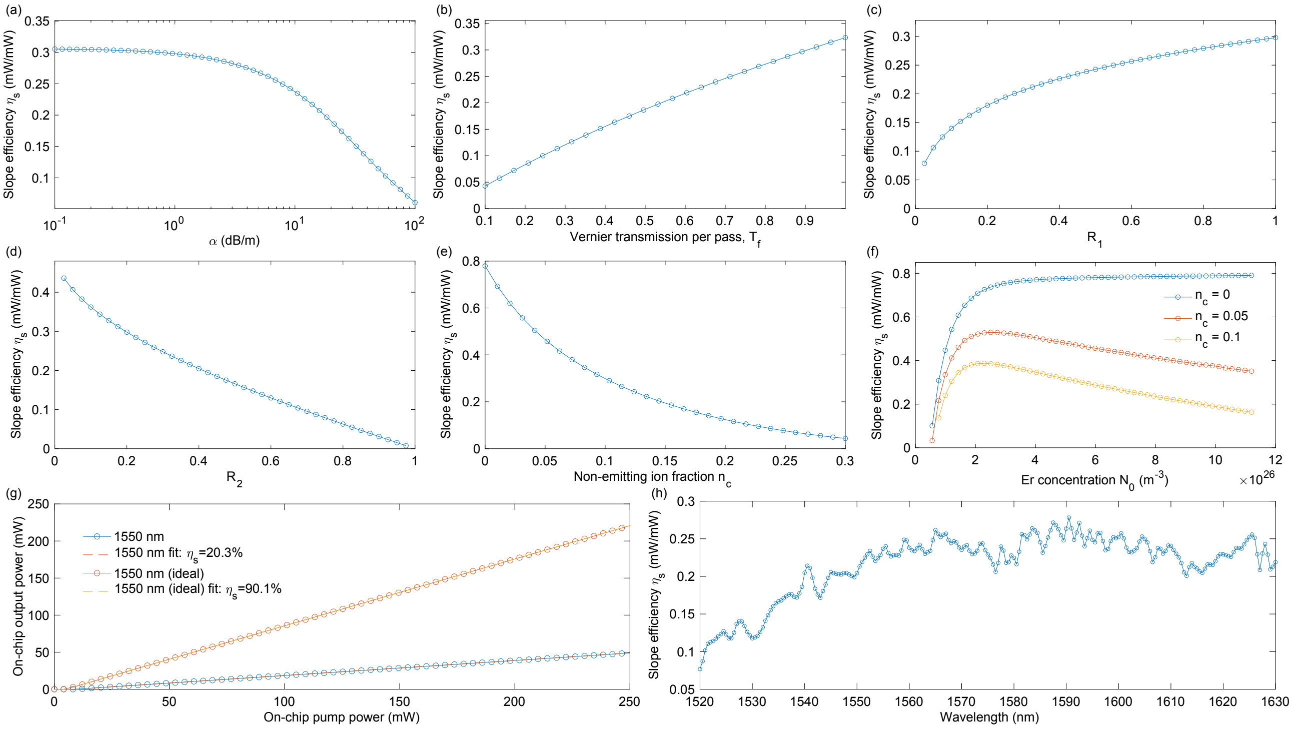

Supplementary Figure S9. **Parametric sensitivity of the slope efficiency η_s at 1550 nm.** (**a**) Slope efficiency as a function of passive loss α (dB/m) with Vernier filter transmission T_f=0.9, R_1=1, R_2=0.2, non-emitting (parasitic) fraction n_c=0.1. The pump loss is fixed to 1 dB/m. (**b**) Slope efficiency as a function of Vernier transmission T_f, α=1 dB/m, R_1=1, R_2=0.2, n_c=0.1. (**c**) Slope efficiency as a function of R_1, with α=1 dB/m, T_f=0.9, R_2=0.2, n_c=0.1. (**d**) Slope efficiency as a function of R_2, with α=1 dB/m, T_f=0.9, R_1=1, n_c=0.1. (**e**) Slope efficiency as a function of non-emitting ion fraction n_c, with α=1 dB/m, T_f=0.9, R_1=1, R_2=0.2. (**f**) Slope efficiency as a function of Er concentration N_0, with α=1 dB/m, T_f=0.9, R_1=1, R_2=0.2, for n_c=0, 0.05 and 0.1. (**a**–**f**) assume the implanted Er concentration N_0=5.6×10^{26} m^{-3}. (**g**) Simulated output-pump characteristic for extraction of on-chip slope efficiency η_s=dP_{out}/dP_{pump} at 1550 nm, using measured wavelength-dependent passive loss and Vernier transmission extracted from the device (loop mirrors R_1=1, R_2=0.2, n_c=0.1, N_0=5.6×10^{26} m^{-3}) and ideal values in simulation (α=1 dB/m, T_f=1, R_1=1, R_2=0.2, n_c=0, N_0=5.6×10^{26} m^{-3}). The slope efficiency is obtained from a linear fit to data points above threshold. (**h**) Simulated wavelength dependent slope efficiency using measured loss and Vernier filter transmission. Fluctuations arise from variation from fitted external coupling rate in test resonators.

the stimulated processes. At steady state, $\frac{dN_2}{dt} = \frac{dN_1}{dt} = 0$, the population inversion at point z is:

$$n(z) = \frac{N_2}{N_1 + N_2} = \frac{\tau\sigma_{s,12}\frac{I_s}{h\nu_s} + \tau\sigma_{p,12}\frac{I_p}{h\nu_p}}{\tau\left(\sigma_{s,12} + \sigma_{s,21}\right)\frac{I_s}{h\nu_s} + \tau\left(\sigma_{p,12} + \sigma_{p,21}\right)\frac{I_p}{h\nu_p} + 1} \tag{7}$$

For a given pump, the forward and backward powers are propagated with passive loss to obtain the pump-only inversion $n(z)$ for threshold evaluation: The round-trip small-signal factor $G_0 = R_1 R_2 T_f^2 \exp\left[2\int\left(g_{\text{eff}}(z) - \alpha\right)dz\right]$ determines threshold (Eq. 5), where $g_{\text{eff}}(z) = \Gamma\left(n(z)\left(1 - n_c\right)\sigma_{21} - \left(\left(1 - n(z)\right)\left(1 - n_c\right) + n_c\right)\sigma_{12}\right)N_0$ is the effective gain at position z, and $n_c = 1 - \frac{N_1 + N_2}{N_0}$ denotes the fraction of non-emitting erbium ions arising from pair-induced quenching at high Er concentrations [10], a mechanism likely present in our lasers. Above threshold ($G_0 > 1$), the forward and backward signal and pump are propagated as:

$$\frac{dI(z)}{dz} = g(z)I(z) - \alpha I(z) \tag{8}$$

with boundary conditions $P_+(0) = R_1 P_-(0)$ and $P_-(L) = R_2 P_+(L)$. The inversion in Eq. 7 is updated from intensities $I_s(z) = \Gamma_s\left[P_+(z) + P_-(z)\right]/A_{s,\text{eff}}$ and $I_p(z) = \Gamma_p\left[P_{p,\text{fwd}}(z) + P_{p,\text{bwd}}(z)\right]/A_{p,\text{eff}}$ until convergence, while the left boundary is enforced by bisection on $P_+(0)$. In the fabricated device, mirror 1 is WDM-like, whereas in the simulations we deliberately approximate mirror 1 as a broadband reflector identical to mirror 2, with 80% pump reflectivity, to model an effectively single-sided-pumped cavity with enhanced pump recycling. The Vernier filter transmission is applied once per pass. The right-port output is $P_{out} = (1 - R_2)\,P_+(L)$.

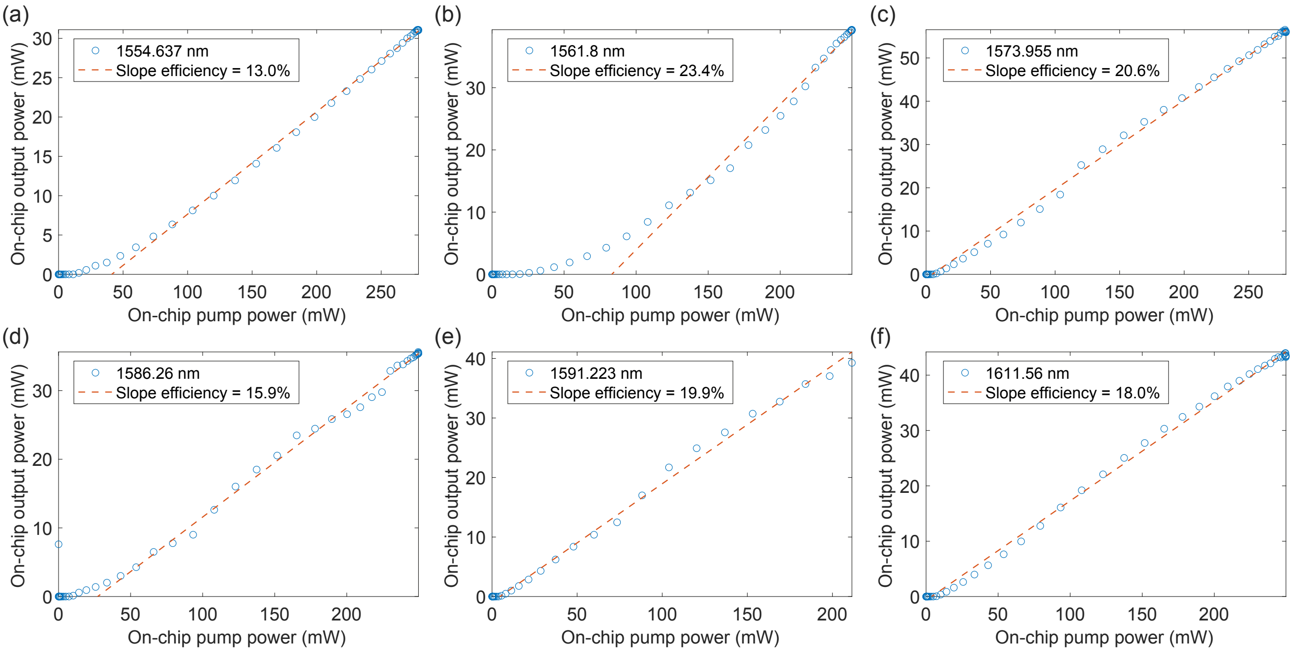

Supplementary Figure S10. **Measured slope efficiency of the EDWL at different wavelengths.**

Supplementary Figure S7(a) presents a schematic of the laser cavity, comprising a high-reflectivity back mirror (reflection R_1), an output coupling mirror (reflection R_2), an erbium-doped gain section, and a mode filter with transmission T_f. The pump light is injected backward, at the interface between the gain section and the Vernier filter (Fig. 2A in the main text).

Supplementary Figure S7(b) shows the simulated intracavity power distribution, where mirror transmissions and filter losses are highlighted. The forward-propagating field initially decreases, reaches a minimum, and then rises toward the end of the Er-doped gain section, while a similar trend is observed for the backward-propagating field. This behavior arises from the pumping scheme in Supplementary Figure S7(a): In the backward pumped configuration, $g(z)$ is initially small or negative and increases along z; thus, the forward wave $P_+(z)$ attenuates in the weakly inverted region before amplifying where $g(z) > 0$. Conversely, the backward wave $P_-(z)$ grows near the pumped end and decays as it propagates into regions of lower inversion, reaching its maximum where $g(z) = 0$.

Using Eq. 6 and Eq. 8, we simulate the output power P_out and its dependence on passive propagation loss α, loop-mirror reflectivities R_1 and R_2, Vernier-filter transmission $\mathrm{T_f}$, Er-ion clustering ratio n_c, Er concentration N_0, and gain length, at a fixed on-chip pump power of 200 mW.

Simulations in Supplementary Figure S8 show that output power is governed by several cavity parameters: P_out rises with lower propagation loss α and higher Vernier transmission T_f. Maximizing the reflection of mirror 1 ($R_1 \to 1$)

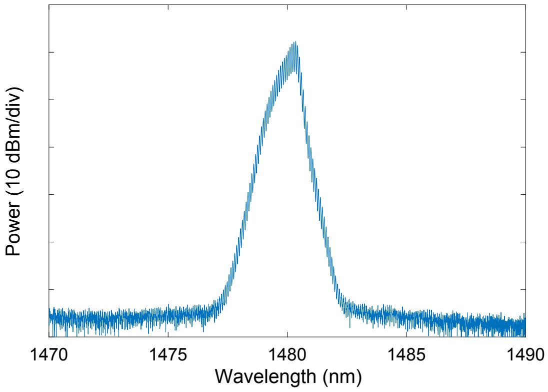

Supplementary Figure S11. **Optical spectrum of the 1480 nm pump laser** (QPhotonics QFBGLD-1480-500).

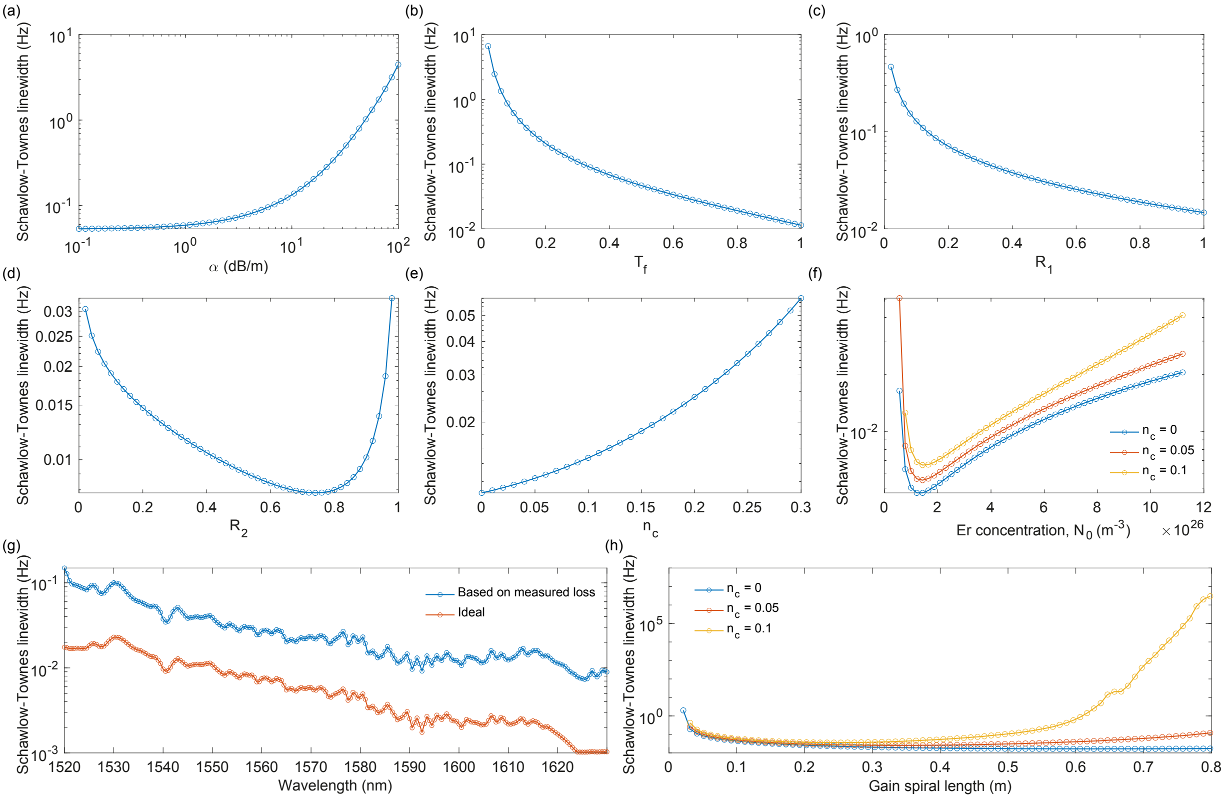

Supplementary Figure S12. **Parametric sensitivity of Schawlow-Townes linewidth.** (**a**) Schawlow-Townes linewidth as a function of passive loss α (dB/m) with Vernier filter transmission T_f=0.9, R_1=1, R_2=0.2, non-emitting (parasitic) fraction n_c=0.1. The pump loss is fixed to 1 dB/m. (**b**) Schawlow-Townes linewidth as a function of Vernier filter transmission T_f, α=1 dB/m, R_1=1, R_2=0.2, n_c=0.1. (**c**) Schawlow-Townes linewidth as a function of R_1, with α=1 dB/m, T_f=0.9, R_2=0.2, n_c=0.1. (**d**) Schawlow-Townes linewidth as a function of R_2, with α=1 dB/m, T_f=0.9, R_1=1, n_c=0.1. (**e**) Schawlow-Townes linewidth as a function of non-emitting ion fraction n_c, with α=1 dB/m, T_f=0.9, R_1=1, R_2=0.2. (**f**) Schawlow-Townes linewidth as a function of Er concentration N_0, with α=1 dB/m, T_f=0.9, R_1=1, R_2=0.2, for n_c=0, 0.05 and 0.1. (**a**–**f**) assume the implanted Er concentration N_0=5.6×10^{26} m^{-3}. (**g**) Simulated wavelength-dependent Schawlow-Townes linewidth, based on measured passive loss and Vernier transmission from fabricated devices (blue trace, loop mirrors: R_1=1, R_2=0.2, n_c=0.1, N_0=5.6×10^{26} m^{-3}), comparing with a constant propagation loss of 1 dB/m and a Vernier filter transmission of 0.9 (red trace). (**h**) Schawlow-Townes linewidth as a function of gain spiral length, based on measured passive loss and Vernier transmission from fabricated devices, showing an optimum around 0.08–0.1 m at an Er concentration of N_0=5.6×10^{26} m^{-3}, with n_c varying from 0 to 0.1.

monotonically increases power by reducing round-trip loss, while the output mirror R_2 exhibits a low-reflectivity optimum, where relatively weak output coupling maximizes power by balancing extraction with intracavity build-up. We observe that even a small non-emitting fraction n_c strongly suppresses power, motivating mitigation of Er clustering. At fixed 17 cm gain length, lowering the Er concentration raises output by reducing clustering (Supplementary Figure S8(f)); at a fixed Er concentration of $N_0 = 5.6\times10^{26}$ m^{-3}, the optimal gain length is $\sim$ 8 cm (Supplementary Figure S8(h)). Including the measured wavelength dependences of α, T_f, and Er cross sections ($\sigma_{s,12}$ and $\sigma_{s,21}$), the model reproduces the experimentally observed lower C-band power relative to the L-band. In an idealized device ($n_\mathrm{c} = 0$, $\alpha(\lambda) = 1$ dB/m, $T_\mathrm{f} = 1$), the predicted P_out is nearly flat over the Er emission band (Supplementary Figure S8(g)).

The slope efficiency η_s is obtained from a linear fit of P_out versus pump power just above threshold.

The dominant levers for higher slope efficiency η_s are minimizing Vernier insertion loss, using high R_1 and low R_2, suppressing clustering (small n_c), and selecting an optimal N$_0$, similar to the ones of P_out. Supplementary Figure S9(h) shows the simulated slope efficiency in the 1520–1630 nm range, revealing that slope efficiency peaks at wavelengths longer than the Er emission maximum ($\sim$ 1535 nm): Near the C-band the required transparency inversion is high because both absorption and emission cross sections are large; much of the pump is therefore spent merely overcoming reabsorption (including from the non-emitting pool), which depresses η_s. Toward longer wavelengths $\sigma_{12}(\lambda)$ falls faster than $\sigma_{21}(\lambda)$ so reabsorption weakens and the incremental signal gain per pump photon increases. Meanwhile, the passive waveguide loss $\alpha(\lambda)$ decreases at longer wavelengths, enhancing internal efficiency through higher Vernier transmission and reduced pump dissipation. Consequently, the simulation predicts a broad maximum in η_s around $\sim$ 1570–1600 nm.

We measured the EDWL slope efficiency at various wavelengths (Supplementary Figure S10) to validate the simulation in Supplementary Figure S9(h), observing the same trend of reduced efficiency and higher threshold pump power near 1550 nm.

To further increase the slope efficiency, a spectrally narrow 1480 nm pump should be used to maximize overlap with the Er^{3+} $^4I_{15/2} \rightarrow {}^4I_{13/2}$ absorption and to minimize off-resonant pump power [11]. Our 1480 nm pump has a 3 dB spectral width of $\sim$ 1 nm (Supplementary Figure S11), frequency components detuned from the peak are absorbed with lower cross-section. A narrower-band 1480 nm pump would increase the effective absorption coefficient and inversion in our EDWL, and thus can further improve the EDWL slope efficiency under otherwise identical power conditions.

Alternatively, a 980 nm pump can be used to further improve the efficiency due to its higher absorption cross section and reduced excited-state absorption in erbium, leading to more efficient population inversion. Our current 1480 nm pumping scheme is a compromise due to the edge-coupler design and propagation loss.

Using Eq. 6 and Eq. 8, we simulate the Schawlow-Townes laser linewidth: $\Delta\nu_{\mathrm{ST}} = n_{\mathrm{sp}} \frac{h\nu}{4\pi P_{\mathrm{out}}} (\Delta\nu_c)^2$, where $n_{\mathrm{sp}} = \frac{N_2}{N_2+N_1}$ is the population inversion factor, $\Delta\nu_c = \frac{1}{2\pi\tau_{\mathrm{ph}}}$ is the cavity loss rate, with τ_{ph} the photon lifetime.

A similar dependence of the minimum Schawlow-Townes linewidth on α, mirror reflectivities R_1, R_2, Vernier filter transmission T_{f}, and the non-emitting fraction n_{c} is observed as for the output power. As shown in Supplementary Figure S12(g), longer wavelengths with reduced reabsorption yield narrower Schawlow-Townes linewidths in simulation. In experiments, however, the laser frequency noise is dominated over most offset frequencies by technical sources such as TRN and mechanical coupling.

Supplementary Note 7. Frequency noise transduction from thermorefractive noise in Vernier ring resonators

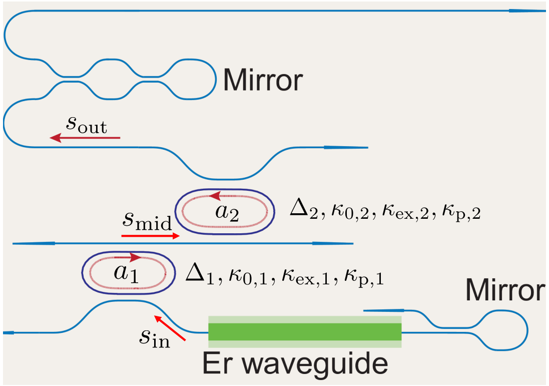

Supplementary Figure S13. **Schematic illustrating the analysis of transduction from TRN in Vernier ring resonators to laser frequency noise.**

Thermorefractive noise (TRN) is the fundamental refractive index fluctuation in a dielectric caused by thermodynamic temperature fluctuations of the material. In a microresonator, random, equilibrium fluctuations δT within the optical mode change the refractive index via the thermo-optic coefficient $\mathrm{d}n/\mathrm{d}T$, thereby jittering the cavity resonance frequency ω_0.

To evaluate the contribution of TRN in Vernier ring resonators, we consider a double-resonator model, as illustrated in Supplementary Figure S13, and analyze its transmission characteristics. The Langevin equations for the system can be written as follows [12, 13]:

$$\frac{\mathrm{d}a_1}{\mathrm{d}t} = -i\Delta_1 a_1 - \kappa_1 a_1 + \sqrt{\kappa_{\mathrm{ex},1}} s_{\mathrm{in}} \tag{9}$$

$$\frac{\mathrm{d}a_2}{\mathrm{d}t} = -i\Delta_2 a_2 - \kappa_2 a_2 + \sqrt{\kappa_{\mathrm{ex},2}} s_{\mathrm{mid}} \tag{10}$$

and the coupling relation:

$$s_{\mathrm{mid}} = -\sqrt{\kappa_{\mathrm{ex},1}} a_1, \; s_{\mathrm{out}} = -\sqrt{\kappa_{\mathrm{ex},2}} a_2 \tag{11}$$

where $s_{\rm in}$, $s_{\rm mid}$ and $s_{\rm out}$ are the complex field amplitudes at various points within the laser cavity, and $|s|^2$ corresponds to the photon flux at each respective position. For resonator j, $a_{\rm j}$ represents the resonator mode, with $|a_{\rm j}|^2$ denoting the photon number within the resonator. $\kappa_{\rm 0,j}$, $\kappa_{\rm ex,j}$, $\kappa_{\rm p,j}$ and $\kappa_{\rm j}$ represent, respectively, the intrinsic loss, coupling strength to the fundamental mode, parasitic loss, and total loss. $\kappa_{\rm j} = \frac{\kappa_0}{2} + \kappa_{\rm ex,j} + \kappa_{\rm p,j}$ $(j = 1, 2)$. Finally, $\Delta_{\rm j} = \omega_{\rm j} - \omega$ denotes the detuning of the laser from the cavity mode, where ω is the lasing frequency.

Starting from equation 9, 10 and 11, we derive the relationship between $s_{\rm in}$ and $s_{\rm out}$:

$$\Rightarrow s_{\rm out} = \frac{\kappa_{\rm ex,1}}{i\Delta_1 + \kappa_1}\frac{\kappa_{\rm ex,2}}{i\Delta_2 + \kappa_2}s_{\rm in} \tag{12}$$

The phase relationship is given by:

$$\varphi_{\rm vernier} = \arg\left(\frac{s_{\rm out}}{s_{\rm in}}\right) = \tan^{-1}\left(\frac{\Im[s_{\rm out}/s_{\rm in}]}{\Re[s_{\rm out}/s_{\rm in}]}\right) = -\tan^{-1}\frac{\Delta_1}{\kappa_1} - \tan^{-1}\frac{\Delta_2}{\kappa_2} \tag{13}$$

For the longitudinal mode within the laser cavity, the round-trip phase must be an integer multiple of 2π:

$$\varphi_{\rm rt} = 2\varphi_{\rm vernier} + 2\beta L_{\rm cav} = 2\pi m, m \in \mathbb{N} \tag{14}$$

where $\beta = \frac{n_{\rm eff}\omega}{c}$ is the effective propagation constant and $L_{\rm cav}$ is the single-pass cavity length. We introduce a perturbation to the round-trip phase, and the resulting variance must be zero:

$$\delta\varphi_{\rm rt} = \frac{\partial\varphi_{\rm rt}}{\partial\omega}\delta\omega + \frac{\partial\varphi_{\rm rt}}{\partial\omega_1}\delta\omega_1 + \frac{\partial\varphi_{\rm rt}}{\partial\omega_2}\delta\omega_2 = 0 \tag{15}$$

The derivatives are expressed as follows:

$$\frac{\partial\varphi_{\rm rt}}{\partial\omega} = \frac{2/\kappa_1}{1 + (\Delta_1/\kappa_1)^2} + \frac{2/\kappa_2}{1 + (\Delta_2/\kappa_2)^2} + \frac{2L_{\rm cav}}{c}n_{\rm g} \tag{16}$$

$$\frac{\partial\varphi_{\rm rt}}{\partial\omega_1} = -\frac{2/\kappa_1}{1 + (\Delta_1/\kappa_1)^2}, \ \frac{\partial\varphi_{\rm rt}}{\partial\omega_2} = -\frac{2/\kappa_2}{1 + (\Delta_2/\kappa_2)^2} \tag{17}$$

To maximize transduction and avoid underestimating the influence of TRN, we set $\Delta_1 = \Delta_2 = 0$. For our Vernier ring design, we use $\kappa_1 \approx \kappa_2 \approx 15\ \kappa_0$, with $\kappa_0 \approx\ 40\ \text{MHz} \cdot 2\pi$. Given laser cavity length $L_{\rm cav} \approx 20$ cm and effective group index $n_{\rm g} \approx 1.8$, we obtain:

$$\delta\omega = \frac{\delta\omega_1 + \delta\omega_2}{2 + L_{\rm cav}\kappa_1 n_{\rm g}/c} \approx \frac{\delta\omega_1 + \delta\omega_2}{6} \tag{18}$$

Therefore, the relationship between the laser frequency noise (FN) and the frequency fluctuations of the Vernier rings due to TRN can be expressed as follows:

$$S_{\delta \rm f} = \frac{S_{\delta \rm f_1} + S_{\delta \rm f_2}}{36} \approx \frac{S_{\delta \rm f_1}}{18} \tag{19}$$

where $S_{\delta \rm f_1}$ is the frequency noise power spectral density of a single Vernier ring due to TRN. In microresonators, the frequency noise PSD relates to temperature fluctuations by $S_{\delta \rm f} = \left(f_0 \frac{{\rm d}f}{{\rm d}n}\frac{{\rm d}n}{{\rm d}T}\right)^2 S_{\delta \rm T} \propto T^2$ [14]. In the approximation of Eq. 19, we assume the two Vernier rings to have the same temperature at 400 K, although this slightly overestimates the transduced frequency noise, as in experiments one of the rings is maintained at room temperature.

In our EDWL, spectral hole burning can occur in the ground and $^4I_{13/2}$ manifolds. However, because the Er-doped gain waveguide operates near room temperature, any hole burned by the intracavity field is shallow and rapidly refilled owing to the broad homogeneous linewidth and ultrafast Stark-level equilibration, manifesting only as minor local gain compression at the lasing line. As the Vernier filter determines the lasing wavelength and strongly suppresses adjacent modes, such weak hole burning neither shifts the lasing wavelength nor induces mode hopping, and it does not limit the tuning range.

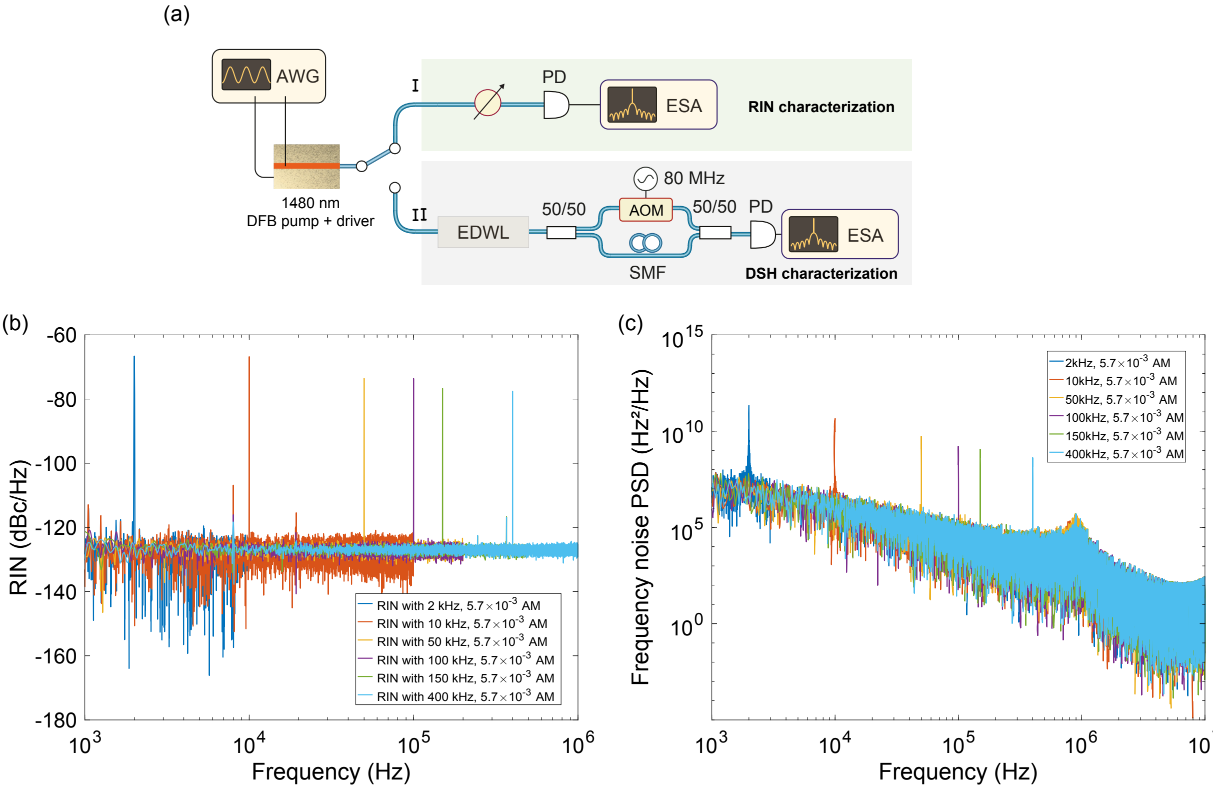

Supplementary Figure S14. **Pump RIN and frequency noise transduction.** (**a**) Experimental setup for the pump RIN to the EDWL frequency noise transduction. An AWG applies a sinusoidal current $i(t) = I_{\text{bias}} + \Delta I \sin(2\pi f_{\text{m}} t)$. The two paths: (I) measures the pump RIN with a photodiode and an electrical spectrum analyzer. The EDWL output (II) is analyzed by delayed self-heterodyne interferometry. (**b**) Pump RIN measurement for modulation index $m = \frac{\Delta I}{I_{\text{bias}}} = 5.7 \times 10^{-3}$ at $f_m = 2, 10, 50, 100, 150$, and 400 kHz. An AC-coupled pre-amplifier is inserted between the PD and ESA. (**c**) The corresponding EDWL frequency noise spectral density from DSHI. Discrete peaks show the RIN to FN transduction.

Supplementary Note 8. Frequency noise transduction from pump laser RIN

We quantified the transduction from pump-laser intensity modulation to the EDWL frequency noise [15] using the setup in Supplementary Figure S14(a). An arbitrary waveform generator (AWG) applied a small sinusoidal current to the 1480-nm pump diode driver:

$$i(t) = I_{\text{bias}} + \Delta I \sin(2\pi f_{\text{m}} t) \tag{20}$$

with $I_{\text{bias}} = 1400$ mA. The driver's RF input has a modulation transconductance $C_{\text{mod}} = 200$ mA/V and a bandwidth of 1.2 MHz. For an AWG setting $V_{\text{pp}} = 40$ mV, the current amplitude $\Delta I = \frac{1}{2} C_{\text{mod}} V_{\text{pp}} = 4$ mA. The calibrated fractional modulation is $m_{\text{pp}} = 2\Delta I / I_{\text{bias}} \approx 5.7 \times 10^{-3}$. With low modulation depth and within the modulation bandwidth, the relationship between current modulation and laser frequency noise was assumed to be linear. This was verified by the absence of harmonic peaks on the RIN spectrum after applying the modulation.

At a few modulation frequencies ν from 2 kHz to 400 kHz, we measured both the pump RIN and laser frequency noise (Supplementary Figure S14(b)(c)). The transduction function $H(\nu)$ is calculated as follows:

$$H(\nu) = \frac{\int_{\text{peak}} S_{\delta\nu}(\nu') d\nu'}{\int_{\text{peak}} \text{RIN}(\nu') d\nu'} \tag{21}$$

where the integration is performed over the peaks at modulation frequency ν in the laser frequency noise power spectral density and pump RIN. The transduction function exhibits a linear relation on a log-log scale (Supplementary Figure S15(a)).

As shown in Supplementary Figure S15(c), the transduced frequency noise was determined by multiplying the pump RIN without modulation (Supplementary Figure S15(b)) by the transduction function $H(\nu)$:

$$S_{\delta\nu}(\nu) = H(\nu) \cdot \text{RIN}(\nu) \tag{22}$$

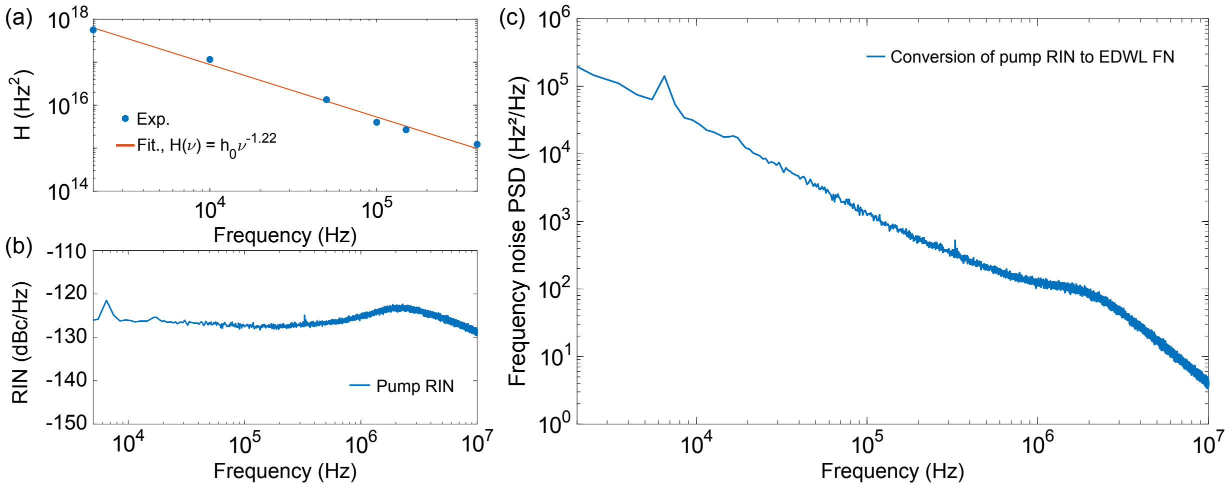

Supplementary Figure S15. **Transduction function and the computed noise contribution.** (**a**) Measured and fitted transduction function $H(\nu)$ according to equation 21. (**b**) Measured relative intensity noise (RIN) of the 1480 nm laser diode. (**c**) Calculated laser frequency noise transduced from the pump RIN.

Supplementary Note 9. Wafer-scale uniformity of the EDWL tunability and frequency noise

We evaluated wafer-level reproducibility by testing EDWLs from nine stepper fields (F1–F9) on a 4-inch wafer. The lithography layout is a 5 × 5 array of 2.024 cm × 2.024 cm fields. The nine central fields were fully exposed and selected for characterization. Each EDWL was tuned by heating the Vernier microrings and was unidirectionally pumped at 1480 nm with ∼380 mW off-chip power.

Supplementary Figure S16 summarizes the results: Supplementary Figure S16(a)-(i) show C+L-band tuning spectra that are qualitatively consistent across all fields, indicating reproducible wafer-scale fabrication and high functional yield. Variations in span are attributed primarily to ∼1.2% Si_3N_4 thickness non-uniformity across the wafer, which perturbs the Vernier-ring FSRs and the attainable loop-mirror reflectivity range. Additional variation arises from fiber-to-chip coupling drift that changes the on-chip pump power. Implementing bidirectional pumping, tightening LPCVD Si_3N_4 thickness control (<0.6%), and adopting a more fabrication-tolerant mirror (e.g., a looped MZI with intentional arm imbalance) would further equalize the tuning span.

Supplementary Figure S16(j) reports the EDWL frequency-noise spectra (heterodyne measurement with a low-noise Toptica ECDL, setup in Fig. 3A in the main text) for a representative device in field F7 measured from 1548 to 1619 nm. The spectra exhibit comparable low-offset behavior and a common intrinsic floor $S_{\delta\nu}$ <100 Hz^2/Hz above 1 MHz. The relaxation-oscillation peak shifts slightly with instantaneous output power. Supplementary Figure S16(k) shows frequency noise at 1559 nm for devices from F1–F9, revealing no systematic dependence on field location.

Collectively, these results demonstrate wafer-scale reproducibility of the EDWL performance.

Supplementary Note 10. EDWL frequency noise response to external injection and self-reflection

In this section, we present the EDWL frequency-noise measurements as a function of the reflection level for external injection and for self-reflection (Figs. S17(a)-(c)).

With external injection detuned by less than 217 MHz (Supplementary Figure S17(d)), the DSH spectrogram remains unchanged across the tested powers up to 10 dBm (Supplementary Figure S17(a)), and the corresponding frequency-noise spectra show no systematic linewidth degradation (Supplementary Figure S17(g)). This stability can be explained by the Vernier filter's microresonator drop ports, which function as intrinsic bandpass filters and inherently suppress back-reflections.

In contrast, increasing self-reflection produces a progressive reduction of low-offset frequency noise; the integrated linewidth decreases monotonically and, at the highest return (full self-reflection), approaches that of a tabletop ECDL used as the local oscillator (Supplementary Figure S17(h)). We observe that, at strong self-reflection (<15 dB attenuation), the EDWL intermittently switches between a noise-broadened state and a narrow-line coherent

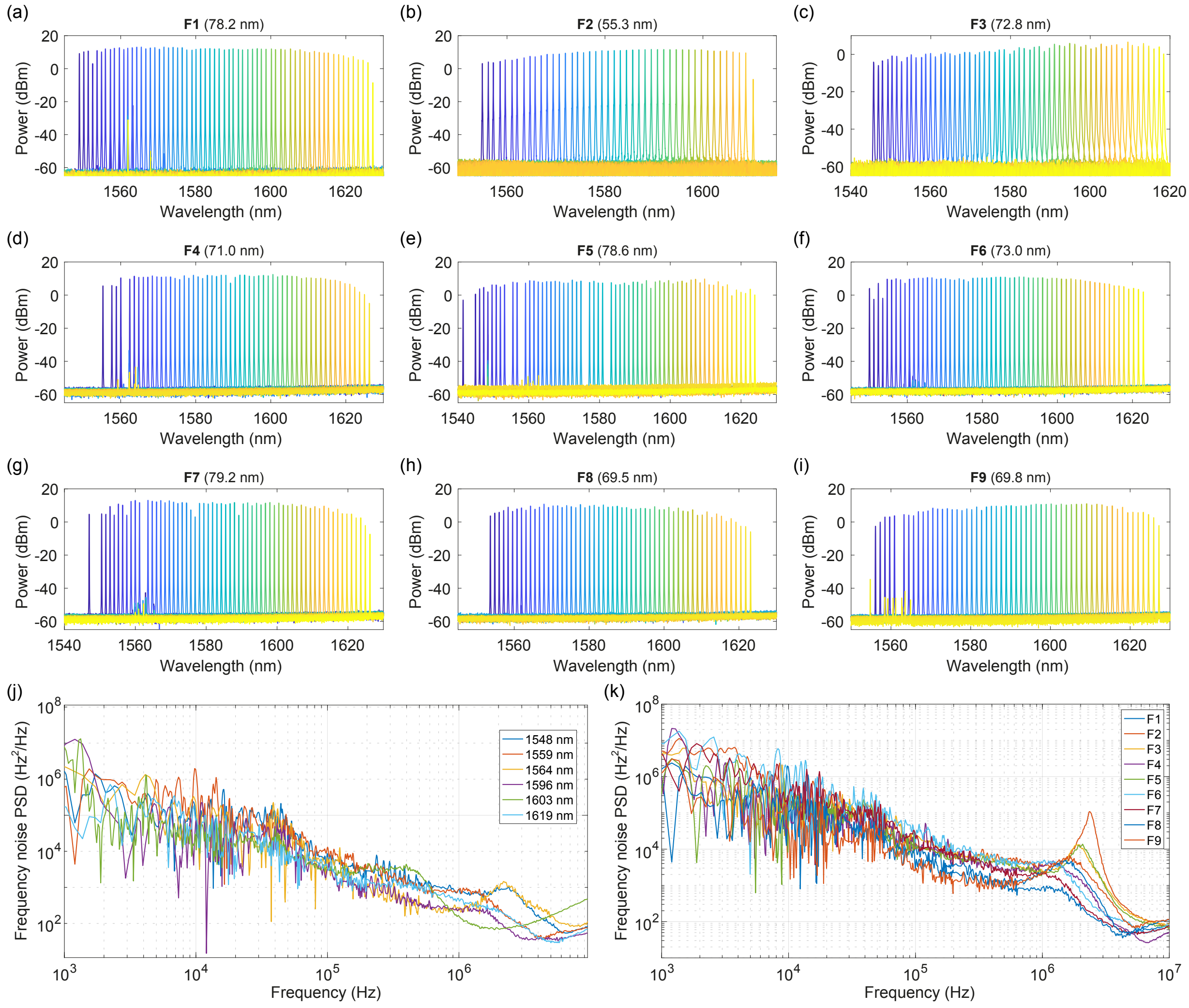

Supplementary Figure S16. **Wafer-scale characterization of the EDWL tunability and frequency noise.** (**a**)-(**i**) Tuning spectra for EDWLs from stepper fields F1–F9 with unidirectional pumping (∼380 mW off-chip). (**j**) Frequency-noise spectra of the EDWL (F7) from 1548 nm to 1619 nm, showing comparable low-offset noise and a similar intrinsic noise floor; relaxation-oscillation peaks shift with output-power variation. (**k**) Frequency-noise spectra at 1559 nm for devices from F1–F9.

state, which are visible in the DSH spectrum (Supplementary Figure S17(b)) and beat-note traces (Supplementary Figure S17(e)(f)). This bistability is consistent with delayed self-injection in a compound cavity described by the Lang-Kobayashi model: slow drifts of feedback phase (and polarization-dependent return) move the operating point between stable external-cavity solutions and neighboring external-cavity modes [16, 17]. Because the broadened state is non-stationary and non-Lorentzian, a linewidth is not well defined; we therefore report linewidths for the coherent state.

The observed linewidth narrowing with increasing self-reflection follows the expected action of coherent optical negative feedback. The reinjected field is a time-delayed copy of the intracavity field; for offset frequencies below the delay corner $f_c \sim \frac{1}{2\pi\tau}$ [18, 19], the feedback acts as passive phase regulation, yielding a closed-loop suppression factor that, in linearized form, scales as $\left|1 + Ge^{-j2\pi f\tau}\right|^{-2}$ [16, 17]. This mechanism and the associated linewidth-narrowing relations under weak feedback are well established in the cited literature.

In summary, the linewidth reduction in our EDWL at increasing self-reflection is different from ECDLs, which have a large linewidth-enhancement factor α_{H} and are very sensitive to uncontrolled optical feedback. In ECDLs with added reflection, the intensity and phase coupling often drive coherence collapse. Thus, ECDLs use optical isolation to suppress feedback and stabilize single-frequency operation. In our EDWL, the frequency noise reduces monotonically with increased self-reflection. Part of the observed frequency-noise reduction arises from an increased effective photon lifetime due to the external reflection (extended effective cavity), together with delayed self-injection that provides negative feedback on phase fluctuations and suppresses low-frequency frequency noise.

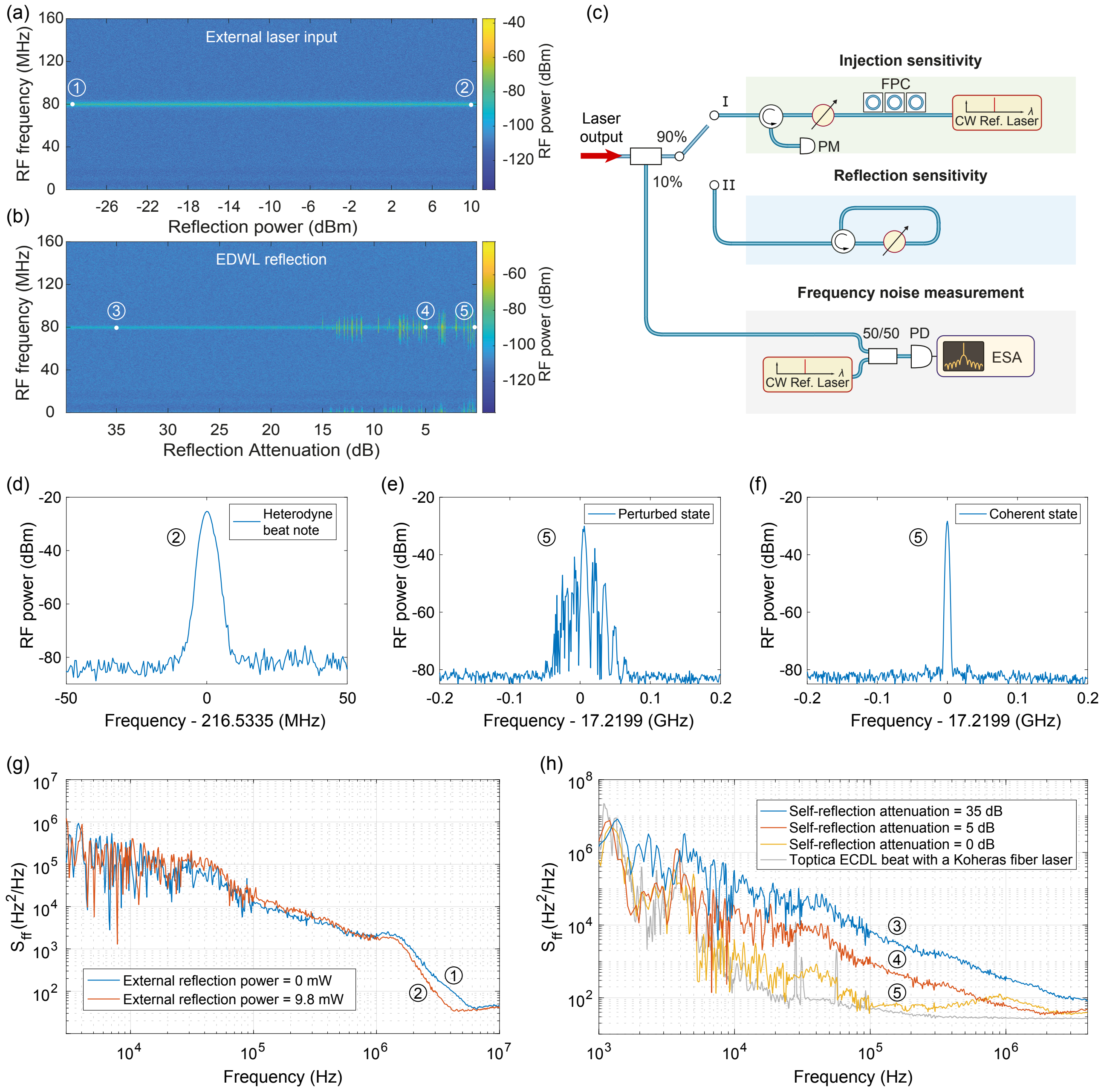

Supplementary Figure S17. **Frequency noise response of the EDWL to external injection and self-reflection.** (**a**) Spectrogram of the delayed self-heterodyne (DSH) beatnote while increasing the injected external-laser power. (**b**) Spectrogram of the DSH beatnote while increasing the self-reflection (return via circulator and VOA; higher return at lower attenuation). (**c**) Experimental setup for frequency noise measurement with external and internal injection. (**d**) Optical heterodyne beatnote between the EDWL and the injected laser, showing a detuning $\Delta\nu$=216.53 MHz. (**e**) Heterodyne beatnote between the EDWL and a Toptica ECDL used as the local oscillator (LO) at 0 dB self-reflection attenuation, showing a noise-broadened (perturbed) state. (**f**) A coherent (narrow-line) state at 0 dB self-reflection attenuation. (**g**) EDWL frequency noise spectra for two external-injection points marked in (**a**): state 1 (0 mW) and state 2 (9.8 mW). (**h**) EDWL frequency noise for three self-reflection levels marked in (**b**): state 3 (35 dB), state 4 (5 dB), and state 5 (0 dB), showing progressive low-offset noise reduction with increasing return. The LO's own frequency-noise spectrum (Toptica ECDL, measured against a Koheras fiber laser) is shown in grey for reference. At the coherent state with full self-reflection, the EDWL frequency noise approaches that of the tabletop ECDL.

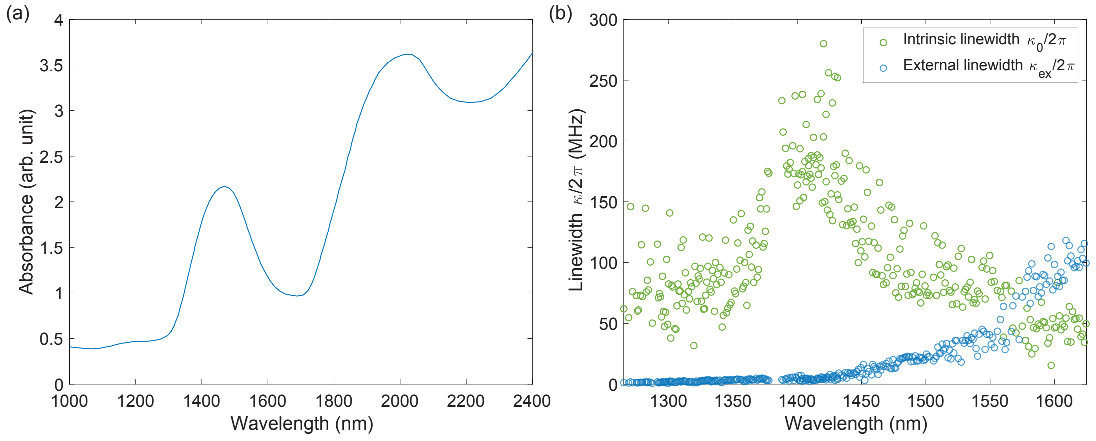

Supplementary Figure S18. **O-H absorption from residual H_2O in Si_3N_4 PICs.** (**a**) Near-infra-red absorption spectrum of water [20]. (**b**) Broadband linewidth measurement of Si_3N_4 resonators with O-H related absorption.

Supplementary Note 11. Hydrogen-related absorption and Er^{3+} quenching in Si_3N_4 PICs

Hydrogen-related bonds (N-H, Si-H) intrinsic to LPCVD Si_3N_4 increase loss in the 1500–1530 nm band [21] but can be largely eliminated by long-duration high-temperature annealing [1]. When Si_3N_4 is cladded with SiO_2, residual water from wet cleaning can be trapped in confined features (e.g., submicron bus-ring gaps) and, if not adequately removed, gives rise to additional O-H absorption in the same spectral range (Supplementary Figure S18(a), [20]) and suppresses C-band output in our EDWL. This O-H-related absorption was observed in test resonators with small features. In Supplementary Figure S18(b), we quantify the wavelength-dependent passive loss via broadband intrinsic-linewidth measurements of a Si_3N_4 microring (5 μm × 200 nm) with SiO_2 cladding and a 1 μm bus-ring gap. The intrinsic linewidth $\kappa_0(\lambda)$ extracted from resonance fits was converted to propagation loss (dB/m) using $\alpha = 10\log_{10}(e) n_g \kappa_0 / c$, yielding a loss at 1500 nm approximately twice that at 1600 nm. This confirms an additional O-H contribution, likely from residual water in the small coupling gap. The excess O-H-related loss is concentrated over ∼ 1500–1550 nm with only a weak long-wavelength tail. The effect occurs only in devices with bus-ring gaps smaller than 1 μm; devices of identical cross-section but larger gaps show no added loss (Fig. 2D in the main text). The EDWL employs gaps larger than 1 μm and should therefore remain unaffected by this absorption.

It is well established that O-H groups are efficient non-radiative quenchers of Er^{3+} emission near 1.55 μm: the $^4I_{13/2} \rightarrow {}^4I_{15/2}$ energy gap can be bridged by one O-H overtone or two fundamentals, shortening the Er^{3+} lifetime and lowering quantum efficiency [22, 23]. However, such quenching requires near-field coupling, i.e., O-H co-located with Er^{3+} within a few nanometers. In our devices, any O-H, when present, resides primarily in the SiO_2 cladding or as trapped water in small gaps; it therefore contributes to propagation loss rather than Er site quenching.

Supplementary Note 12. Group delay of Vernier resonators

In this section, we quantify the group delay introduced by the Vernier microrings to account for the discrepancy between the geometric round-trip time and that inferred from the measured mode spacing. The EDWL has a geometric round-trip length $L \approx 40.14$ cm. With $n_g \approx 1.8$ at 1550 nm , the corresponding round-trip time is $\tau = n_g L/c \approx 2.41$ ns. The measured free-spectral range ($\Delta\nu \approx 300$ MHz) on the ESA gives $\tau = 1/\Delta\nu \approx 3.33$ ns. The excess $\Delta\tau \approx 0.92$ ns is attributed to the Vernier rings' group delay.

In Section Supplementary Note 7, we derived the phase added by the Vernier rings:

$$\varphi_{\text{vernier}} = -\tan^{-1}\frac{\Delta_1}{\kappa_1} - \tan^{-1}\frac{\Delta_2}{\kappa_2} \tag{23}$$

where $\Delta_\text{j} = \omega_\text{j} - \omega$ are the detunings from each ring resonance and $\kappa_\text{j} = \frac{\kappa_0}{2} + \kappa_{\text{ex,j}} + \kappa_{\text{p,j}}$ denote the decay rates. The additional group delay during each cavity round trip is:

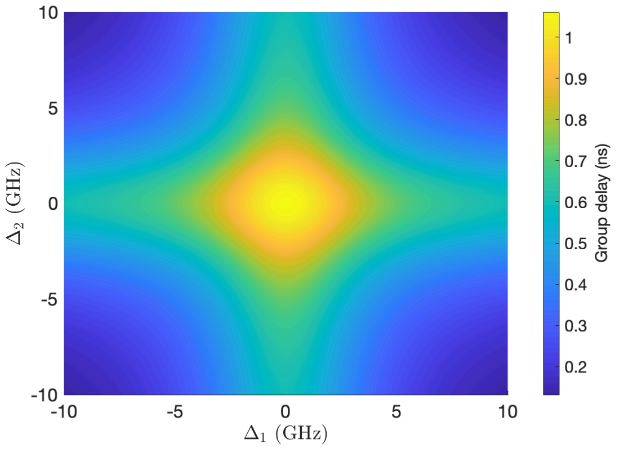

Supplementary Figure S19. **Additional round-trip delay from the Vernier filter as a function of detuning.**

$$\tau_{\text{vernier}} = 2\frac{\mathrm{d}\varphi_{\text{vernier}}}{\mathrm{d}\omega} = \frac{2/\kappa_1}{1+(\Delta_1/\kappa_1)^2} + \frac{2/\kappa_2}{1+(\Delta_2/\kappa_2)^2} \tag{24}$$

Using $\kappa_1 = \kappa_2 \approx 0.6$ GHz $\cdot 2\pi$, the calculated round-trip delay map versus (Δ_1, Δ_2) is shown in Supplementary Figure S19. At dual resonance ($\Delta_1 = \Delta_2 = 0$), the Vernier filter adds a delay of ~ 1 ns to the cavity round-trip time, consistent with the 0.92 ns inferred from the measured mode spacing.

Supplementary Note 13. Effect of heater resistivity drift on laser frequency stability

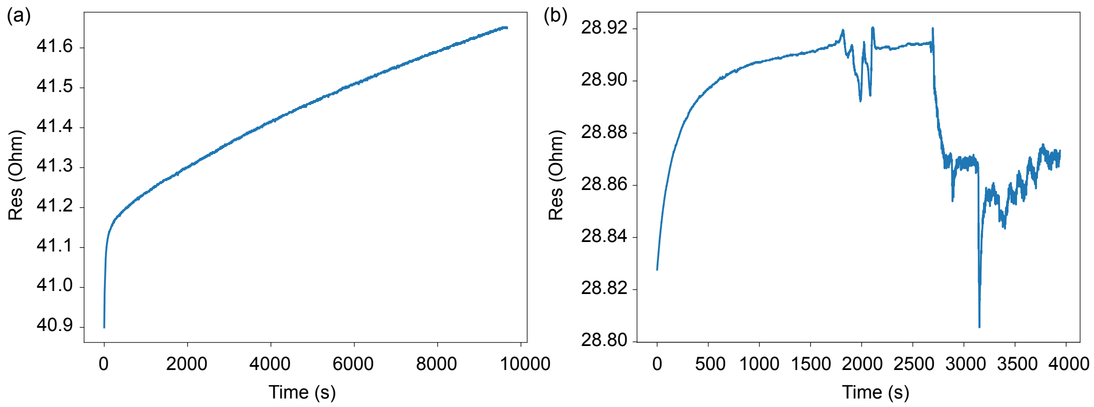

Supplementary Figure S20. **Micro-heater resistance variation over time.** (**a**) Heater resistance drift in an isolated environment, illustrating a gradual change over time. (**b**) Heater resistance drift with isolation disruption at 2700 s, showing sudden changes from environmental factors.

This section identifies micro-heater resistance drift as a source of the EDWL frequency drift, influencing lasing frequency through resonance alignment in the Vernier filter and output power adjustment via tunable loop mirrors.

The micro-heaters, with a 5×0.525 μm^2 cross-section, are fabricated through sequential DC sputtering of a 25 nm titanium adhesion layer and a 500 nm platinum layer onto the SiO_2 top cladding. Heaters are formed by direct laser writing onto a 3 μm AZ 10XT photoresist layer, followed by Argon ion beam etching.

After fabricating the micro-heaters, a constant voltage is applied, and the output current is monitored to calculate resistance drift as the voltage-to-current ratio. Supplementary Figure S20(a) shows heater resistance drift in an isolated environment (experimental setup enclosed with a cover) over 2.7 hours, exhibiting a continuous upward trend. In contrast, Supplementary Figure S20(b) highlights sudden changes and fluctuations after removing the cover at 2700 s, exposing the micro-heater to ambient conditions.

Resistance changes are common in semiconductor devices [24], particularly in thin-film resistive materials like the micro-heaters used in EDWLs. In metal conductors, gradual resistance changes can arise from oxidation [25] and electromigration [26] caused by constant current flow. Environmental factors such as humidity, contamination, and airflow further exacerbate these effects. Isolating the EDWL from the surrounding environment is therefore critical, and alternative actuators reducing direct resistive heating, for instance AlN or PZT, may help mitigate laser frequency drift.

Supplementary Note 14. Noise figure measurement of erbium-doped waveguide amplifiers

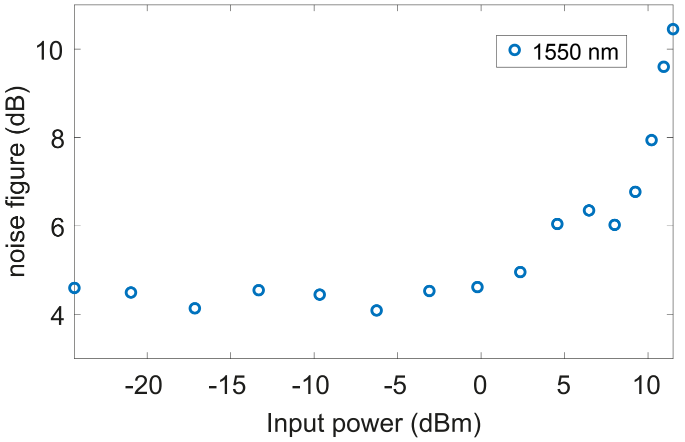

Supplementary Figure S21. **Measured noise figure of the 17-cm-long Er:Si_3N_4 waveguide amplifier under various off-chip input powers at 1550 nm.**

We measured the noise figure (NF) of a stand-alone 17 cm Er:Si_3N_4 EDWA (same cross-section as used in the EDWL) using the standard source-subtraction method [27] at 1550 nm under bidirectional 1480 nm pumping, with 330 mW off-chip power on each side. The fiber-to-fiber insertion loss was 2.5 dB. The measurement results are presented in Supplementary Figure S21.

In the high-gain regime (weak inputs), the system NF is approximately 4–5 dB; as the input increases and the amplifier saturates, the NF rises. These values include coupling and passive losses; reducing those losses and operating closer to full inversion ($n_{sp} \rightarrow 1$) moves the intrinsic EDWA NF into the EDFA class. Because signal reabsorption decreases toward longer wavelengths, the NF is expected to be lower in the L-band [28].

The long erbium upper-state lifetime ($\tau_{21} \approx 3.4$ ms) passively filters pump fluctuations, so only low-frequency pump noise can appreciably modulate gain; our pump RIN (-120 to -130 dBc/Hz, Supplementary Figure S15(b)) makes this effect negligible in the NF context [3].

For comparison, semiconductor optical amplifiers typically exhibit larger effective n_{sp} and internal loss due to interband gain with fast carrier dynamics, leading to higher NF than erbium-doped amplifiers.

Supplementary Note 15. Footprint considerations and compact layout

The large area of our EDWL ($\sim$40 mm^2) reflects design choices of the 200 nm Si_3N_4 platform used to enable wafer-scale erbium implantation at reduced, standard-implanter-compatible energies. The thinner waveguide core reduces mode confinement, necessitating larger bend radii to suppress radiation loss and wider spiral gaps to mitigate intermodal coupling. The layout in Figure 1D in the main text thus prioritizes low propagation loss and robustness over minimum footprint.

To illustrate footprint scalability without altering the cavity architecture, we designed an alternative compact layout with an area of $\sim$23.8 mm^2 (Supplementary Figure S22(a)). This design preserves the Vernier-filtered cavity, gain-spiral length, and coupling conditions, and is expected to maintain comparable lasing performance while nearly halving the footprint, implying roughly a twofold increase in yield on a given wafer.

As one of the loop mirrors is operated at high reflectivity to minimize cavity loss, it may be replaced by a broadband

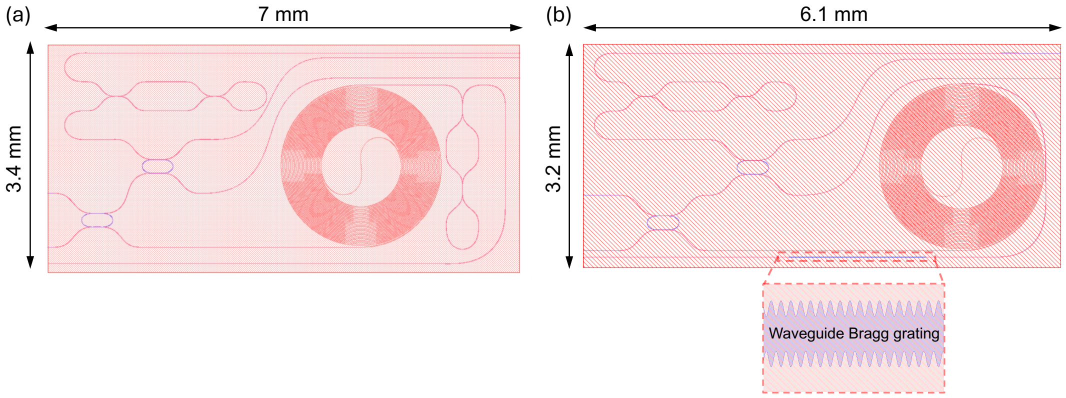

Supplementary Figure S22. **Compact layouts of EDWLs.** (**a**) Schematic of an alternative floorplan ($\sim$23.8 mm^2) that preserves the cavity architecture and gain length while reducing bend count and routing area. (**b**) A broadband waveguide Bragg grating can replace the WDM loop mirror to further compress footprint.

waveguide Bragg grating, which provides near-unity reflection in a more compact form (Supplementary Figure S22(b)) at the expense of a tuning range limited by its finite stopband.

Supplementary Note 16. Lasing in high-Er-concentration EDWLs

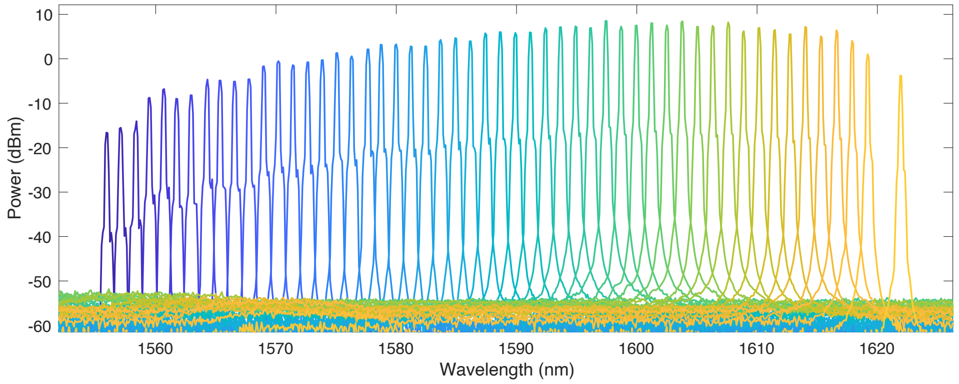

Supplementary Figure S23. **Lasing map of a high-Er concentration EDWL (9.4×10^{26} m^{-3}), showing tunability and reduced C-band power compared to the 5.6×10^{26} m^{-3} device.**

To evaluate the effect of increased erbium concentration, we measured the lasing spectrum of an EDWL with a higher Er content than that in the main text.

Our direct ion implantation yields a Gaussian depth profile with excellent lateral uniformity, suppressing local clustering relative to co-deposition or diffusion methods. Consistent with limited quenching, we measure lifetimes of 3.4 ms at Er peak concentration of 5.6×10^{26} m^{-3} and 2.7 ms at 9.4×10^{26} m^{-3} in Er:Si_3N_4 waveguides. The EDWL performance is dominated by pump depletion and parasitic absorption rather than lifetime shortening.

We characterized an EDWL with a higher total concentration at 9.4×10^{26} m^{-3}. With $\sim$180 mW unidirectional on-chip pump at 1480 nm, it lases across the C+L bands with output power and tuning range comparable to those of devices with 5.6×10^{26} m^{-3} Er concentration (Supplementary Figure S23). A notable difference is a reduced C-band output relative to the lower-concentration device, which is consistent with our simulations of wavelength dependence in the presence of Er ion clustering (Supplementary Figure S8(e)(f)), indicating a stronger concentration-dependent quenching at higher Er doping.

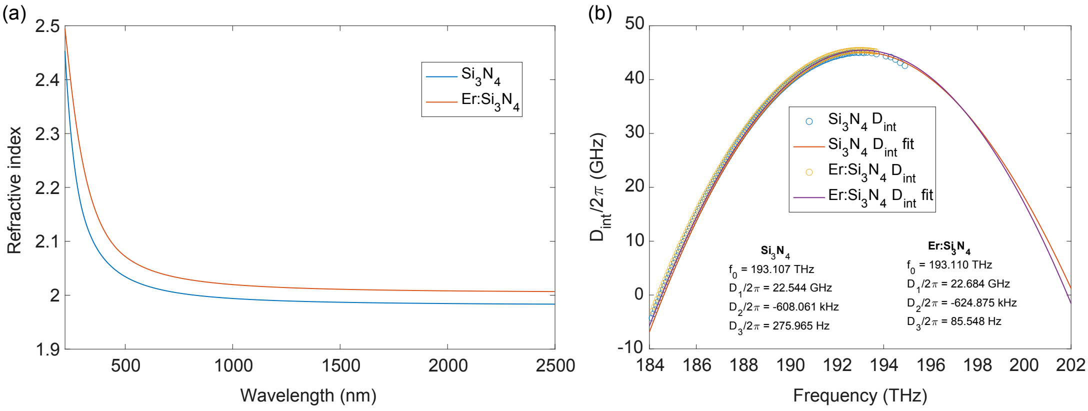

Supplementary Figure S24. **Refractive index modification in Si_3N_4 from Er implantation.** (**a**) Measured refractive index of Si_3N_4 film before and after Er implantation. (**b**) Integrated dispersion $D_{\mathrm{int}}/2\pi$ of a 22.5 GHz ring resonator before and after Er implantation.

Supplementary Note 17. Refractive index modification of Si_3N_4 by Er implantation

To quantify the erbium-induced refractive-index change in Si_3N_4, we performed spectroscopic ellipsometry on blanket films before and after implantation. The implantation sequence consisted of three energy steps (350, 178, and 81.2 keV) with a total dose of 2.63×10^{15} ions/cm^2, corresponding to a peak Er concentration of 3.25×10^{26} ions /m^3 ($\sim 0.34\%$ atomic ratio in Si_3N_4).

Supplementary Figure S24(a) shows the measured refractive indices $n(\lambda)$ before and after implantation. Implanting 0.34% Er increases the film index slightly, from 1.9866 to 2.0106 at 1552 nm, indicating a small modification of material dispersion $|\mathrm{d}n/\mathrm{d}\lambda|$. To assess the optical impact on waveguides, we measured the integrated dispersion $D_{\mathrm{int}}/2\pi$ of a 22.5 GHz Si_3N_4 ring resonator before and after implantation (Supplementary Figure S24(b)). The free spectral range ($D_1/2\pi$) increased from 22.544 GHz to 22.684 GHz, corresponding to a $\sim 0.6\%$ reduction in the group index $n_{\mathrm{g}} = c/(L \cdot \mathrm{FSR})$. This behavior is consistent with the measured index change: although n_{eff} slightly increases, the slope $\mathrm{d}n_{\mathrm{eff}}/\mathrm{d}\lambda$ decreases, resulting in a lower n_{g}. Minor geometry or stress variations from implantation and post-annealing may contribute to this small dispersion shift, but the overall effect remains weak. At higher Er concentrations, a gradual increase in the Si_3N_4 refractive index is expected. Further investigation of the concentration dependence will be reported in future work.

Acknowledgments: The experimental datasets and scripts used to produce the plots in this paper are available at Zenodo (https://doi.org/10.5281/zenodo.18459173).

Supplementary References

[1] X. Ji, R. Ning Wang, Y. Liu, J. Riemensberger, Z. Qiu, and T. J. Kippenberg, Optica **11**, 1397 (2024).
[2] J. Liu, G. Huang, R. N. Wang, J. He, A. S. Raja, T. Liu, N. J. Engelsen, and T. J. Kippenberg, Nature communications **12**, 2236 (2021).
[3] Y. Liu, Z. Qiu, X. Ji, A. Lukashchuk, J. He, J. Riemensberger, M. Hafermann, R. N. Wang, J. Liu, C. Ronning, and T. J. Kippenberg, Science **376**, 1309 (2022).
[4] Y. Liu, Z. Qiu, X. Ji, A. Bancora, G. Lihachev, J. Riemensberger, R. N. Wang, A. Voloshin, and T. J. Kippenberg, Nature Photonics **18**, 829 (2024).
[5] L. Rubin and J. Poate, Industrial Physicist **9**, 12 (2003).
[6] P. Myslinski, D. Nguyen, and J. Chrostowski, Journal of lightwave technology **15**, 112 (1997).
[7] X. Dong, N. Q. Ngo, P. Shum, B.-O. Guan, H.-Y. Tam, and X. Dong, Optics letters **29**, 358 (2004).
[8] W. Jiang, D. Xu, S. Yao, B. Xiong, and Y. Wang, Materials Science in Semiconductor Processing **43**, 222 (2016).
[9] Z. Ye, H. Jia, Z. Huang, C. Shen, J. Long, B. Shi, Y.-H. Luo, L. Gao, W. Sun, H. Guo, *et al.*, Photonics Research **11**, 558 (2023).
[10] E. Delevaque, T. Georges, M. Monerie, P. Lamouler, and J.-F. Bayon, IEEE Photonics Technology Letters **5**, 73 (2002).
[11] E. Desurvire, C. Giles, J. R. Simpson, and J. Zyskind, Optics letters **14**, 1266 (1989).
[12] H. A. Haus, *Waves and fields in optoelectronics* (Prentice-Hall, 1984).
[13] M. L. Gorodetsky and V. S. Ilchenko, Journal of the Optical Society of America B **16**, 147 (1999).
[14] G. Huang, E. Lucas, J. Liu, A. S. Raja, G. Lihachev, M. L. Gorodetsky, N. J. Engelsen, and T. J. Kippenberg, Physical Review A **99**, 061801 (2019).
[15] E. Lucas, P. Brochard, R. Bouchand, S. Schilt, T. Südmeyer, and T. J. Kippenberg, Nature communications **11**, 374 (2020).
[16] R. Lang and K. Kobayashi, IEEE journal of Quantum Electronics **16**, 347 (1980).
[17] J. Mork, B. Tromborg, and P. L. Christiansen, IEEE journal of quantum electronics **24**, 123 (2002).
[18] M. Lipka, M. Parniak, and W. Wasilewski, Applied Physics B **123**, 238 (2017).
[19] Y.-X. Chao, Z.-X. Hua, X.-H. Liang, Z.-P. Yue, C. Jia, L. You, and M. K. Tey, Physical Review Applied **23**, L011005 (2025).
[20] T. Afrin, S. N. Karobi, M. M. Rahman, M. Y. A. Mollah, and M. A. B. H. Susan, Journal of Solution Chemistry **42**, 1488 (2013).
[21] M. H. Pfeiffer, J. Liu, A. S. Raja, T. Morais, B. Ghadiani, and T. J. Kippenberg, Optica **5**, 884 (2018).
[22] A. Monguzzi, M. Trioni, R. Tubino, A. Milani, L. Brambilla, and C. Castiglioni, Synthetic metals **159**, 2410 (2009).
[23] S. Shen, A. Jha, E. Zhang, and S. J. Wilson, Comptes rendus. Chimie **5**, 921 (2002).
[24] J.-P. Colinge and C. A. Colinge, *Physics of semiconductor devices* (Springer Science & Business Media, 2005).
[25] N. Cabrera and N. F. Mott, Reports on progress in physics **12**, 163 (1949).
[26] K.-N. Tu, Journal of applied physics **94**, 5451 (2003).
[27] D. Derickson, Fiber optic test and measurement/edited by Dennis Derickson. Upper Saddle River (1998).
[28] E. Desurvire, Applied optics **29**, 3118 (1990).
