# Supplementary material for: Wafer-scale manufacturing of ultra-broadband, high-power erbium-doped integrated lasers
Source: Nat Commun. 2026 Mar 10;17:3722. doi: 10.1038/s41467-026-69787-1 (PMC13102962; doi:10.1038/s41467-026-69787-1)
Supplement: Supplementary file 2 — Reporting Summary [file 41467_2026_69787_MOESM2_ESM.pdf]

nature research

Corresponding author(s): Tobias J. Kippenberg

Lasing Reporting Summary

Nature Research wishes to improve the reproducibility of the work that we publish. This form is intended for publication with all accepted papers reporting claims of lasing and provides structure for consistency and transparency in reporting. Some list items might not apply to an individual manuscript, but all fields must be completed for clarity.

For further information on Nature Research policies, including our data availability policy, see Authors & Referees.

ü Experimental design

Please check: are the following details reported in the manuscript?

1. Threshold

Plots of device output power versus pump power over a wide range of values indicating a clear threshold

- [x] Yes
- [ ] No

Figure 3E and Supplementary Figure S10.

2. Linewidth narrowing

Plots of spectral power density for the emission at pump powers below, around, and above the lasing threshold, indicating a clear linewidth narrowing at threshold

- [ ] Yes
- [x] No

This analysis was not included because linewidth narrowing at threshold was not a focus of the manuscript. Our work concentrates on steady-state lasing performance, tunability, and frequency-noise behavior rather than transient spectral evolution across threshold.

Resolution of the spectrometer used to make spectral measurements

- [x] Yes
- [ ] No

Figure 3C and Figure 3D caption.

3. Coherent emission

Measurements of the coherence and/or polarization of the emission

- [x] Yes
- [ ] No

Coherence was evaluated via frequency noise power spectral density measurements (Fig. 3F).

4. Beam spatial profile

Image and/or measurement of the spatial shape and profile of the emission, showing a well-defined beam above threshold

- [ ] Yes
- [x] No

No dedicated spatial beam profile was recorded. This is not critical for this work, as the laser operates in a guided mode and is collected into a single-mode fiber, ensuring a well-defined spatial output mode above threshold. Therefore, separate near-field or far-field beam imaging was not required for the conclusions of the manuscript.

5. Operating conditions

Description of the laser and pumping conditions
Continuous-wave, pulsed, temperature of operation

- [x] Yes
- [ ] No

The laser and pumping conditions are described in the main text under Figure 3 caption and Section “Laser wavelength tuning and emission coherence”. Specifically: “Erbium ions … are optically excited … by in-band pumping from a 1480 nm laser diode (QPhotonics QFBGLD-1480-500, >1 nm spectral width near 1480 nm) with 400 mW nominal power at 1.5 A driving current”.

Threshold values provided as density values (e.g. W cm^{-2} or J cm^{-2}) taking into account the area of the device

- [ ] Yes
- [x] No

Threshold is given as on-chip pump power (mW). While a power density could be defined using the guided mode area, this is not conventional for integrated waveguide lasers, and threshold is commonly reported as coupled pump power. We therefore did not provide density-normalized values.

6. Alternative explanations

Reasoning as to why alternative explanations have been ruled out as responsible for the emission characteristics
e.g. amplified spontaneous, directional scattering; modification of fluorescence spectrum by the cavity

- [ ] Yes
- [x] No

Alternative explanations such as amplified spontaneous emission or cavity-modified fluorescence were not explicitly discussed; however, they are implicitly ruled out by the observation of a clear lasing threshold, high SMSR, single-frequency operation, and coherent beatnote and frequency-noise measurements, which are inconsistent with ASE or spontaneous emission.

7. Theoretical analysis

Theoretical analysis that ensures that the experimental values measured are realistic and reasonable
e.g. laser threshold, linewidth, cavity gain-loss, efficiency

- [x] Yes
- [ ] No

Yes. Supplementary Notes 6-8 provide theoretical modelling using rate equations and noise analysis to compare calculated threshold, slope efficiency, and linewidth with experimental results. Good agreement between simulation and measurement confirms that the reported values are realistic and physically consistent.

8. Statistics

Number of devices fabricated and tested	☒ Yes ☐ No	Devices were fabricated across a 4-inch wafer, and EDWLs from nine stepper fields (F1-F9) were experimentally tested and characterized. Wafer-scale measurement data are provided in Supplementary Note 9 and referenced in the main text, confirming uniform tuning ranges and comparable frequency-noise performance across fields.
Statistical analysis of the device performance and lifetime (time to failure)	☐ Yes ☒ No	Although no statistical lifetime analysis was performed, device stability is evaluated in Fig. 4E, showing continuous lasing operation for over 6 hours with less than 15 MHz frequency drift, indicating stable performance.
